# 3D-QSAR Design of New Bcr-Abl Inhibitors Based on Purine Scaffold and Cytotoxicity Studies on CML Cell Lines Sensitive and Resistant to Imatinib

**DOI:** 10.3390/ph18060925

**Published:** 2025-06-19

**Authors:** David Cabezas, Thalía Delgado, Guisselle Sepúlveda, Petra Krňávková, Veronika Vojáčková, Vladimír Kryštof, Miroslav Strnad, Nicolás Ignacio Silva, Javier Echeverría, Christian Espinosa-Bustos, Guido Mellado, Jiao Luo, Jaime Mella, Cristian O. Salas

**Affiliations:** 1 Instituto de Química, Facultad de Ciencias, Universidad de Valparaíso, Valparaíso 2360102, Chile; david.cabezas@postgrado.uv.cl (D.C.); jiao.luo@postgrado.uv.cl (J.L.); 2 Departamento de Química Orgánica, Facultad de Química y de Farmacia, Pontificia Universidad Católica de Chile, Santiago de Chile 7820436, Chile; tdelgado@uc.cl (T.D.); gsepulvedas@uc.cl (G.S.); nsilvas@estudiante.uc.cl (N.I.S.); 3 Department of Experimental Biology, Palacký University, Slechtitelu 27, 77900 Olomouc, Czech Republicveronika.vojackova@upol.cz (V.V.); vladimir.krystof@upol.cz (V.K.); 4 Laboratory of Growth Regulators, Palacký University and Institute of Experimental Botany, The Czech Academy of Sciences, Slechtitelu 27, 77900 Olomouc, Czech Republic; miroslav.strnad@upol.cz; 5 Departamento de Ciencias del Ambiente, Facultad de Química y Biología, Universidad de Santiago de Chile, Santiago de Chile 9170022, Chile; javier.echeverriam@usach.cl; 6 Departamento de Farmacia, Facultad de Química y de Farmacia, Pontificia Universidad Católica de Chile, Santiago de Chile 7820436, Chile; ccespino@uc.cl; 7 Departamento de Ingeniería Informática, Facultad de Ingeniería, Universidad Católica de Temuco, Temuco 4780000, Chile; gmellado@uct.cl; 8 Centro de Investigación Desarrollo e Innovación de Productos Bioactivos (CInBIO), Escuela de Química y Farmacia, Facultad de Farmacia, Universidad de Valparaíso, Valparaíso 2360102, Chile

**Keywords:** 3D-QSAR, chronic myeloid leukaemia, Bcr-Abl inhibitors, purine derivatives, docking studies, molecular dynamics

## Abstract

**Background/Objectives:** Bcr-Abl inhibitors such as imatinib have been used to treat chronic myeloid leukemia (CML). However, the efficacy of these drugs has diminished due to mutations in the kinase domain, notably the T315I mutation. Therefore, in this study, new purine derivatives were designed as Bcr-Abl inhibitors based on 3D-QSAR studies. **Methods:** A database of 58 purines that inhibit Bcr-Abl was used to construct 3D-QSAR models. Using chemical information from these models, a small group of new purines was designed, synthesized, and evaluated in Bcr-Abl. Viability assays were conducted on imatinib-sensitive CML cells (K562 and KCL22) and imatinib-resistant cells (KCL22-B8). In silico analyses were performed to confirm the results. **Results:** Seven purines were easily synthesized (**7a**–**g**). Compounds **7a** and **7c** demonstrated the highest inhibition activity on Bcr-Abl (IC_50_ = 0.13 and 0.19 μM), surpassing the potency of imatinib (IC_50_ = 0.33 μM). **7c** exhibited the highest potency, with GI_50_ = 0.30 μM on K562 cells and 1.54 μM on KCL22 cells. The GI_50_ values obtained for non-neoplastic HEK293T cells indicated that **7c** was less toxic than imatinib. Interestingly, KCL22-B8 cells (expressing Bcr-Abl^T315I^) showed greater sensitivity to **7e** and **7f** than to imatinib (GI_50_ = 13.80 and 15.43 vs. >20 μM, respectively). In silico analyses, including docking and molecular dynamics studies of Bcr-Abl^T315I^, were conducted to elucidate the enhanced potency of **7e** and **7f**. Thus, this study provides in silico models to identify novel inhibitors that target a kinase of significance in CML.

## 1. Introduction

Leukaemia is a type of blood cancer that is characterized by the uncontrolled proliferation of white blood cells that exhibit an altered morphology and functionality. This phenomenon arises because of a series of mutations that are expressed during the differentiation of these cells into leukocytes [1,2]. In chronic myeloid leukaemia (CML), approximately 95% of patients exhibit a genetic abnormality referred to as the Philadelphia chromosome (Ph) [3]. This abnormality arises from a translocation event between chromosomes 9 and 22 which results in the creation of the Bcr-Abl fusion oncogene. Bcr-Abl is a protein tyrosine kinase that is aberrantly activated and plays a role in the signalling pathway that regulates cell division [4]. Consequently, Bcr-Abl has emerged as a significant therapeutic target for CML, with imatinib being the first-line treatment [5]. Approximately one-third of patients encounter treatment failure with this medication, largely because of point mutations in Bcr-Abl. These mutations can modify the binding of the inhibitor or lead to conformational changes in the enzyme [6]. Over 80 mutations that encode amino acid substitutions that confer varying levels of imatinib resistance have been identified [7]. These mutations can affect residues that directly interact with imatinib. One of the most common mutations is the substitution of isoleucine for threonine at position T315 (T315I), which is found in 2–20% of cases of CML. This mutation causes the enzyme to remain in the active conformation rather than the inactive conformation required for imatinib binding [7]. These findings have led to the development of more potent second-generation inhibitors such as dasatinib and nilotinib. However, like imatinib, they exhibit limited efficacy against certain Bcr-Abl mutations and present other adverse side effects [4,8]. Currently, the only approved third-generation inhibitor is ponatinib, which is specifically designed to overcome the T315I mutation [9].

**Figure 1:**
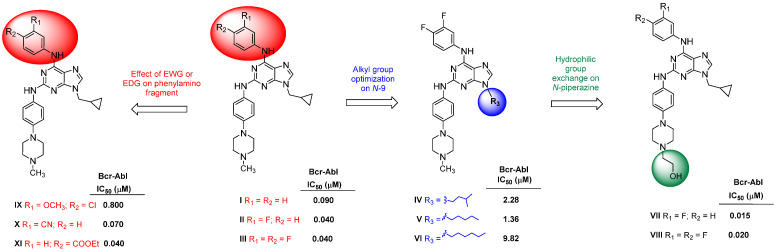
The above chemical structures of purine derivatives with Bcr-Abl activity were developed by our group [11,12,13].

Owing to the limitations of the Bcr-Abl inhibitors that are used clinically, numerous studies have focused on developing novel inhibitors that exhibit reduced side effects or maintain their efficacy in mutated proteins [8,10]. The most common Bcr-Abl inhibitors are those that bind directly to the ATP-binding site, which has resulted in the identification of the purine scaffold as a potential new inhibitor [10]. In this context, our research group has recently designed, synthesized, and assessed a small library of purine derivatives that are modified at the 2, 6, and 9 positions and which can inhibit Bcr-Abl with varying degrees of potency [11,12,13]. Compounds **I**–**III** (as shown in Figure 1) exhibited notable inhibition of Bcr-Abl, with IC_50_ values between 0.040 and 0.090 μM in the Abl kinase inhibition assay [13]. Moreover, compound **III** displayed a higher selectivity for Bcr-Abl compared to other tyrosine kinases, such as BTK, and a serine/threonine kinase, CDK-2. It also demonstrated low micromolar cytotoxicity across various leukaemia cell lines and effectively diminished the phosphorylation of downstream proteins within the Bcr-Abl signalling pathway [13]. Additionally, the optimal substituent at *N*-9 was identified as the cyclopropylmethyl group found in compounds **I**–**III**, which was evidenced by the elevated IC_50_ values of compounds **IV**–**VI**, which possess longer hydrophobic substituents. This conclusion was supported by molecular docking studies which correlated with the dimensions of the hydrophobic pocket in Bcr-Abl [12].

Following this, we synthesized new 2,6,9-trisubstituted purines that were derived from our earlier Bcr-Abl inhibitors, with compound **VII** (see Figure 1) being particularly significant [11]. Compound **VII** demonstrated enhanced potency against Bcr-Abl (IC_50_ = 0.015 μM) in comparison to imatinib and nilotinib and exhibited robust antiproliferative effects on three CML cell lines with Bcr-Abl rearrangement (GI_50_ = 0.7–1.3 μM). Additionally, compound **VII** inhibited the proliferation of KCL22 cells harbouring the Bcr-Abl T315I, E255K, and Y253H point mutations at micromolar concentrations. In contrast, imatinib and nilotinib were ineffective against KCL22 cells with Bcr-Abl^T315I^ (GI_50_ > 20 μM), while **VII**–**VIII** (Figure 1) displayed a GI_50_ ranging from 6.4 to 11.5 μM. Molecular docking studies clarified the structure–activity relationship of these purines concerning Bcr-Abl^WT^ and Bcr-Abl^T315I^. Lastly, cell cycle flow cytometry assays and immunodetection indicated that compound **VII** induced a G1 phase arrest and downregulated downstream Bcr-Abl protein levels in these cells [11]. The influence of certain electron-donating and electron-withdrawing groups (EDG and EWG) in the *para*- and *meta*-positions of the phenylamino fragment at C-6 (compounds **IX**–**XI**, Figure 1) on Bcr-Abl activity has been explored, although definitive conclusions were not reached [12].

Several in silico approaches have been considered for the design of new compounds that can be used as tyrosine kinase inhibitors (TKIs) [14,15,16,17,18,19,20]. QSAR methods have become significant in the development of anticancer drugs due to allowing the prediction of biological activity from molecular structures and thereby aiding in the prioritization of compounds prior to their synthesis. In the context of TKIs, QSAR models allow for a deeper understanding of the structural features that are responsible for their potency and selectivity, guiding the design of more effective analogues with improved pharmacological profiles, as shown by Gond et al. [18]. Likewise, Gagic et al. conducted a review in which they discussed significant studies on the design of novel kinase inhibitors with anticancer applications using methods such as 3D-QSAR, structure-based design, and virtual screening [14]. Urich et al. conducted a de novo study, starting from 84,000 fragments, which were recombined and evaluated through docking to identify new kinase inhibitors [15]. Additionally, mathematical studies have been conducted that used 2D-QSAR with multiple linear regression to identify new PIM1 kinase inhibitors, and their results were validated through molecular dynamics simulations [16]. Moreover, VX-680, an Aurora A kinase inhibitor developed through the use of CoMFA/CoMSIA-based 3D-QSAR models that were combined with molecular docking, exhibited significant potency and in vivo efficacy [21]. Other noteworthy instances include the creation of SYK kinase inhibitors through a blend of pharmacophore modelling, 3D-QSAR, and molecular dynamics, which produced hits with affinities comparable to fostamatinib [22], and the enhancement of p38 MAP kinase inhibitors using CoMFA and CoMSIA [23], which aided in the elucidation of structure–activity relationships within a series of active derivatives. These successful applications underscore the versatility and impact of 3D-QSAR methods in the rational design of selective kinase inhibitors.

Recognizing the potential of in silico molecular modelling techniques to substantially reduce time costs and expenses by efficiently narrowing synthetic optimization options, in this research we utilized our purine derivatives as a basis to develop structure-based 3D-QSAR models. These models were pivotal in designing and synthesizing a small set of new molecules in order to evaluate their inhibition on Bcr-Abl. This study focuses on two main approaches, CoMFA and CoMSIA, which quantitatively evaluate the connections between the molecular polarity/electrostatic characteristics and hydrophobicity of compounds. Furthermore, docking studies were performed on Bcr-Abl^WT^ to interpret the experimental results. The newly synthesized purine derivatives were also tested on imatinib-sensitive and -resistant CML cell lines. Docking and molecular dynamics studies were also conducted for the KCL22-B8 line, which is resistant to imatinib due to its Bcr-Abl^T315I^ mutation.

## 2. Results and Discussion

### 2.1. 3D-QSAR

An in silico study was carried out to organize previously observed structure–activity relationship data and to propose a systematic series of structural modifications that lead to a new family of purines. First, the biological activity (pIC_50_ = −logIC_50_) of a database of 58 purines (Table S1) was correlated with the steric and electrostatic potentials of these purines. The results can be visualized through coloured polyhedra that surround specific fragments of these compounds, which suggests that alterations in the volume and electronic properties of these compounds are necessary to enhance their biological activity. We present our findings in the next section.

#### 2.1.1. Statistical Results

Table 1 summarizes the statistical outcomes of the CoMFA and CoMSIA models. We searched for optimal models using successive field combinations (refer to Table S2). A key parameter for assessing the statistical reliability of a QSAR model is the q^2^ value, which should exceed 0.5. This cross-validation coefficient indicates the internal predictive capability of the QSAR model. For CoMFA, the model that incorporated both field contributions (CoMFA-SE) achieved a value of 0.576. The CoMSIA model that considered steric, electrostatic, and hydrophobic features (CoMSIA-SEH) presented a q^2^ of 0.637. The r^2^_test_ value, which was used to evaluate the external predictive capability of the model displayed in the summary of accepted models in Table 1, was 0.863 for CoMFA-SE, while the CoMSIA-SEH model presented a value of 0.842. The best models also presented a low standard error of the estimate (SEE) and a high non-cross-validation coefficient (r^2^) (0.939 for CoMFA and 0.934 for CoMSIA). The optimal number of components (N) was consistently low across all models (N = 6 for CoMFA and N = 6 for CoMSIA). Although some CoMSIA models reported higher q^2^ values, we chose a model with a greater number of fields to gain more insight into its structure–activity relationship.

Table 2 presents the external validation results for the CoMFA-SE and CoMSIA-SEH models (hereafter referred to as the ‘CoMFA’ and ‘CoMSIA’ models). Both models exhibited high r^2^ test values (0.863 and 0.842, respectively), which indicated satisfactory external predictive capacity. However, as noted by Golbraikh and Tropsha [24,25], while high q^2^ and r^2^ test values (conditions 1 and 2) are essential, they alone do not suffice for model validation. A reliable QSAR model should have a close alignment between predicted and the experimental activity, ideally approximating the line y = x. This was confirmed by meeting conditions [3a or 3b], [4a or 4b], [5a or 5b], and 6, as detailed in Table 2. Condition 7, known as the r^2^m metric, quantitatively assesses the closeness between the observed and predicted activity for the test set. In general, the CoMSIA model displayed better statistical parameters than the CoMFA model.

**Figure 2:**
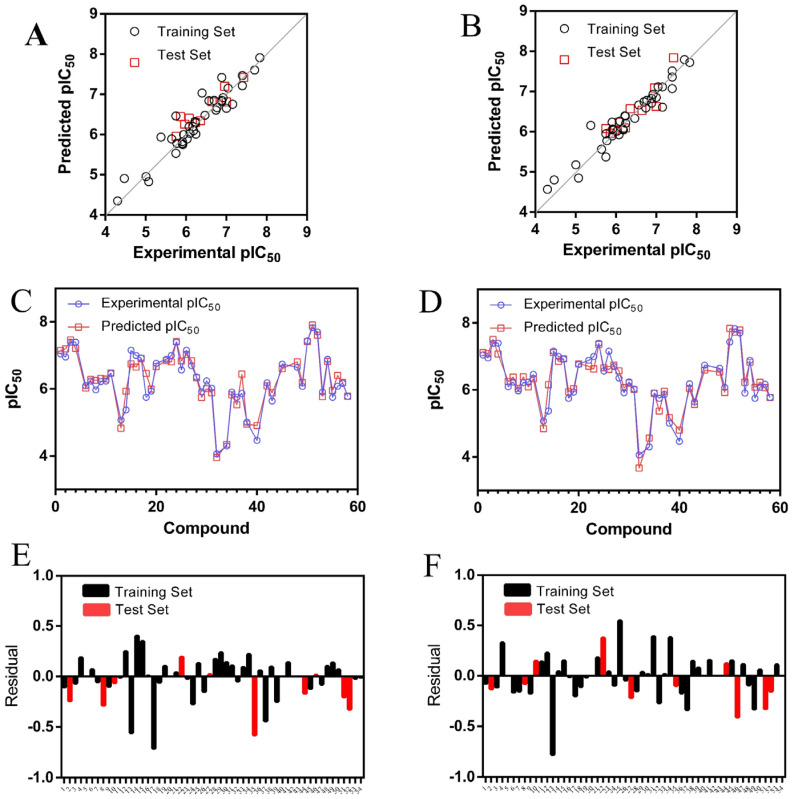
Graphs comparing the experimental and predicted pIC_50_ values for molecules in the training and test sets are shown for the (**A**) CoMFA and (**B**) CoMSIA models. Residual plots are presented for (**C**) CoMFA and (**D**) CoMSIA. Predictions for each molecule in the entire dataset are illustrated for (**E**) CoMFA and (**F**) CoMSIA.

The experimental and predicted activity values along with residuals for the most effective CoMFA and CoMSIA models are displayed in Table S3. All compounds exhibited low residuals with a balanced distribution of positive and negative values. Figure 2A,B depict graphs that compare the experimental and predicted activity, while Figure 2C,D illustrate the residuals and Figure 2E,F show the predictive graphics. The CoMFA and CoMSIA models demonstrated satisfactory predictive capabilities across the entire dataset (training and test sets) and exhibited robust predictive power.

#### 2.1.2. Outliers

In the CoMFA and CoMSIA models, molecules **5**, **21**, **44**, **46**, and **47** were identified as outliers. These compounds produced predictive values with substantial residuals (greater than one logarithmic unit). To evaluate the model’s robustness, a Y-randomization test was performed (Table S4) [26]. The dependent variable (biological activity) was randomly shuffled, and a new QSAR model was generated using the original independent variable matrix. If numerous randomizations yield negative or subpar *q*^2^ and *r*^2^*_ncv_* values (*q*^2^ < 0.5, *r*^2^*_ncv_* < 0.6), it confirms that the results from the final model formulation are not coincidental. In our analysis, the new QSAR models (after several iterations) exhibited low q^2^ and r^2^_ncv_ values (see Table S4).

#### 2.1.3. Applicability Domain

The applicability domain (AD) represents a theoretical region in chemical space that encompasses both the model descriptors and the modelled response. This allows for estimating the uncertainty in predicting a compound based on its similarity to the training compounds used in model development. In this study, we employed the method established by Roy et al. for AD determination [27]. This approach relies on the fundamental principles of standardization. The calculations were conducted using a freely available application on the author’s website, and indicated that all compounds fell within the applicability domain.

#### 2.1.4. Contour Map Analysis

**Figure 3:**
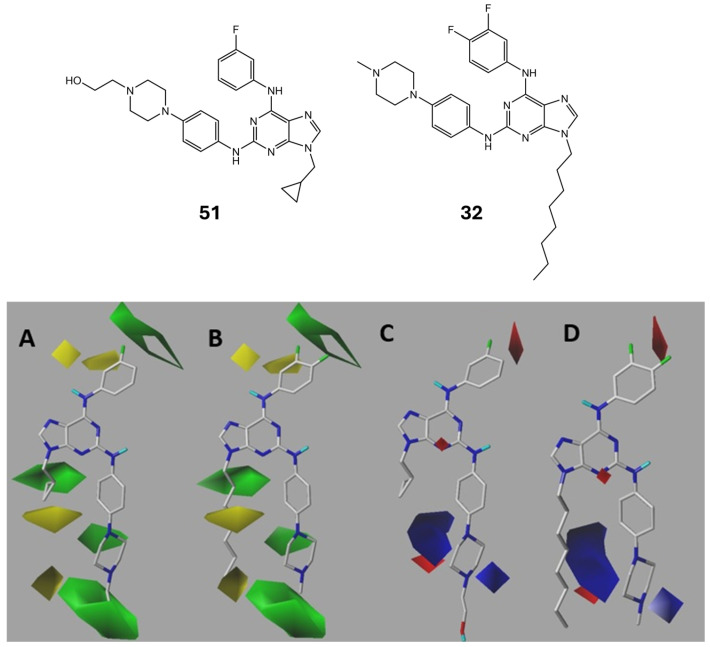
CoMFA steric (**A** for **51** and **B** for **32**) and electrostatic (**C** for **51** and **D** for **32**) contour maps. Green: sterically favoured; yellow sterically disfavoured. Blue: electropositively favoured; red: electronegatively favoured.

**Figure 4:**
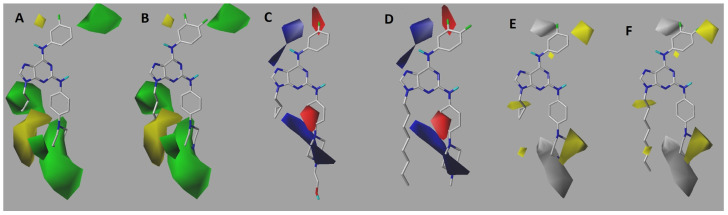
CoMSIA steric (**A** = **51** and **B** = **32**), electrostatic (**C** = **51** and **D** = **32**), and hydrophobic (**E** = **51** and **F** = **32**) contour maps. Green: sterically favoured; yellow: sterically disfavoured (**A**,**B**). Blue: electropositively favoured; red: electronegatively favoured (**C**,**D**). Yellow: hydrophobically favoured; grey: hydrophobically unfavourable (**E**,**F**).

Contour maps represented by coloured polyhedra around the molecules were generated in our study to illustrate the steric, electrostatic, and hydrophobic properties of the molecules. Differently coloured polyhedra indicate regions with favourable or unfavourable molecular characteristics. Figure 3 and Figure 4 showcase the distinct maps for the most active compound (**VII** or **51** according to Table S3, pIC_50_ = 7.833, on the left) and the least active compound (**32**, pIC_50_ = 4.063, on the right).

According to the CoMFA-SE analysis, green and yellow polyhedra are depicted in the steric contour maps. The green polyhedra signify the favourability of high-volume substituents, whereas the yellow polyhedra indicate that bulky compounds are unfavourable. In Figure 3A, the most active molecule is presented, while Figure 3B depicts the least active molecule. The *meta*-position in the aniline fragment was favourable for the presence of bulky groups, whereas the *ortho*-position did not favour such bulky substitutions. Alkylation with a small group at the *N*-9 position is favourable. However, when the carbon chain becomes excessively large (7–8 atoms of carbon), it becomes disavowable. In the electrostatic contour map, red polyhedra signify the favourability of electronegative or electron-rich groups, whereas blue indicates the favourability of electron-deficient groups. A red polyhedron is visible at the *meta*- and *para*-positions of the aminophenyl fragment. This indicates that the electronegative functional groups on the ring are the foundation of its activity. The presence of the piperazine ring is favourable for both steric and electronic activities.

With respect to the contour maps obtained by the CoMSIA method, additional information can be extracted by considering not only the same fields seen in the CoMFA model, but also the hydrophobic fields. Steric contour maps are shown in Figure 4A,B. Overall, the conclusions drawn were similar to those obtained in the CoMFA model, with one difference. This map suggests that bulky substitutions must be present at the *para*-positions of the aniline fragment. Finally, Figure 4E,F show hydrophobic contour maps. The yellow colour indicates favourable hydrophobic substituent activity, whereas the silver-coloured polyhedron suggests favourability for the presence of hydrophilic groups. A yellow polyhedron was observed in piperazine, suggesting that, if this group was to be modified, it should be replaced by a ring with higher lipophilicity or hydrophobicity. Furthermore, a silver polyhedron was apparent in the vicinity of this ring. Therefore, in conjunction with the results of the steric map, the addition of hydrophilic and bulky groups to the ring was suggested. Alkylation at *N*-9 contributes to hydrophobicity. Therefore, cyclopropane was considered favourable. One alternative suggestion is to explore other groups that contribute to hydrophobicity, such as cyclopentane rings. This directly aligns with the conclusions derived from the steric contour maps.

In general, the results obtained from the CoMFA and CoMSIA methods are related to each other. This is conducive to the rational design of new structures.

### 2.2. Design and Synthesis

**Figure 5:**
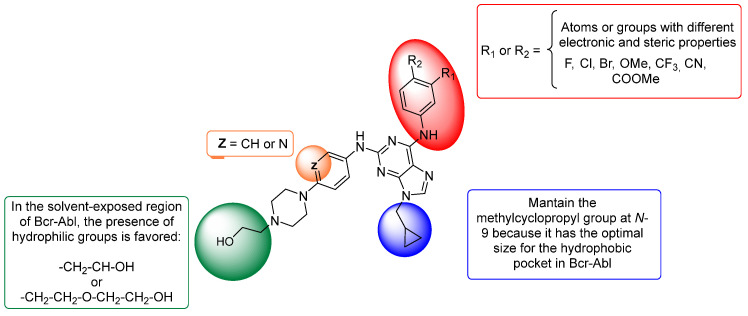
Proposal of novel purine derivatives as potential inhibitors of Bcr-Abl based on 3D-QSAR models.

Based on our previous experimental results and those of the 3D-QSAR study, we used this information to design a small series of seven trisubstituted purine derivatives with different potencies in Bcr-Abl. The proposed structures for the synthesis conducted in this study are shown in Figure 5. To design new purine derivatives, the following is postulated:

(i) Considering the CoMFA and CoMSIA contour maps (green and yellow polyhedra in Figure 3A,B and Figure 4A,B) and by comparing the results of both models for purine **51** (or **VII**, the most active compound of the series, IC_50_ = 0.015 μM), it can be determined that the methylcyclopropyl substituent is much more favourable than the *n*-hexyl substituent (less active compound, **32**, IC_50_ = 86.46 μM). Therefore, it is important to maintain the substitution at *N*-9 with a methylcyclopropyl group (blue region in Figure 5);
(ii) It is important to maintain the hydroxymethyl group attached to piperazine, because it is the most active compound (**VII** and **VIII**, Figure 1), and according to CoMSIA (Figure 4E), the presence of a hydrophilic group favours activity on Bcr-Abl. Likewise, it is interesting to explore the increase in the size of this fragment (green region in Figure 5) because we reported that this fragment is in the solvent-exposed region and is present in dasatinib;
(iii) Based on the CoMSIA analysis, it is proposed that the presence of an electronegative atom in the phenylamino fragment at C-2 of purine would favour activity. That is, the isosteric replacement of benzene by pyridine was considered for these new purine derivatives (orange region in Figure 5);
(iv) In Figure 4C,D, it is observed that the inhibitory activity of Bcr-Abl is stronger in the presence of electron-rich groups or electronegative atoms in the red polyhedra, as well as in the presence of electron-deficient substituents in the blue polyhedra. Thus, the substitution of the fluorine atoms in the *meta*- and/or *para*-positions of the C-6 phenylamino ring by atoms or groups with different electronic and steric properties will be considered (red region in Figure 5). These modifications will allow us to obtain more conclusive information regarding the optimal subtraction patterns of this moiety because this issue is still unclear.

In summary, this design incorporates some chemical modifications at the C-2, *N*-9, and C-6 positions in the purine ring that have not been previously explored by our group, nor have they been considered into other purine derivatives reported as Bcr-Abl inhibitors by other researchers.

**Scheme 1:**
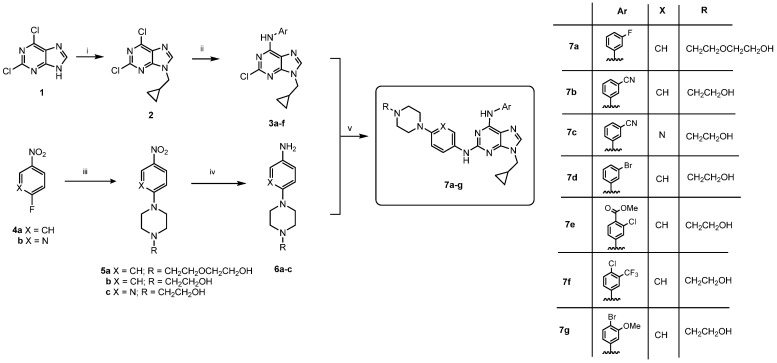
Reagents and conditions: (i) Methylcyclopropyl bromide, K_2_CO_3_, DMF, 12 h, r.t., 60%. (ii) Anilines, DIPEA, *n*-butanol, reflux, 12 h, 50–85%. (iii) 2-(2-(Piperazin-1-yl)ethoxy)ethan-1-ol or *N*-(2-hydroxyethyl)piperazine, K_2_CO_3_, DMF, r.t., 6 h, 93–98%. (iv) H_2_, Pd-C, ethanol, r.t., 4–6 h, quant. (v) K_2_CO_3_ 2M, Pd(OAc)_2_, XantPhos, dioxane, MW, 170 °C, 10 min, 10–52%.

Subsequently, seven designed 2,6,9-trisubstituted purines were synthesized (**7a**–**g**) using the straightforward and efficient synthetic techniques outlined in Scheme 1 and previously reported by our group [11,12,13]. The synthesis began with 2,6-dichloropurine (**1**) as the starting material. Initially, compound **1** was alkylated with methylcyclopropyl bromide under basic conditions, which resulted in compound **2** (excluding its *N*-7 regioisomer) [11,12,13,28,29,30]. To yield **3a**–**f**, regioselective nucleophilic substitution (S_N_Ar) at the C-6 position was performed with the corresponding aniline using *n*-butanol as the solvent and *N*,*N*-diisopropylethylamine as the base. The reaction was carried out under reflux for 12 h to yield the desired intermediates. Following this, a Buchwald–Hartwig C-N coupling reaction at the C-2 of **3a**–**f** with **6a**–**c** was catalysed by palladium (II) and enhanced via microwave irradiation, which yielded the desired compounds **7a**–**g** with yields ranging from 10% to 52%. Aniline derivatives **6a**–**c** were synthesized beforehand from 4-nitro-1-fluorobenzene (**4a**) or 2-fluor-5-nitropyridine (**4b**) through two synthetic steps, as depicted in Scheme 1. Compounds **4a** and **4b** were substituted with piperazine derivatives to form **5a**–**c** (93–98%), which were then quantitatively converted to the necessary aniline derivatives **6a**–**c**. All compounds underwent purification through column chromatography, and their structures were confirmed via spectral analysis (^1^H NMR, ^13^C NMR, and HRMS; see Section 3 and Supplementary Materials).

### 2.3. Kinase Inhibition and Structure–Activity Relationship

To align with this study’s objective of identifying novel Bcr-Abl inhibitors, the next step involved evaluating all synthesized compounds for their kinase inhibition capability. As shown in Table 3, compounds such as **7a**, **7c**, and **7d** displayed enhanced activity relative to imatinib, as evidenced by their lower IC_50_ values (0.13, 0.19, and 0.21 μM compared to 0.33 μM, respectively). From a chemical standpoint, a structure–activity relationship (SAR) can be discerned for this target.

As indicated in Table 3, compound **7a** exhibited the greatest inhibition among all the purine derivatives, achieving an IC_50_ of 0.13 μM on Bcr-Abl. However, it was less effective than its counterpart **VII**, which had an IC_50_ of 0.015 μM. This reduction in activity might be attributed to the elongation of the piperazine-bound 2-hydroxyethyl chain in the C-2-linked aminophenyl segment of the purine. This outcome was anticipated because this alteration was not considered in the purine base utilized in the 3D-QSAR study but was intentionally included in the design to explore this variable for potential future conjugations.

Likewise, from Table 3, it can also be observed that compound **7c** presented an IC_50_ of 0.19 μM in Bcr-Abl, being more potent than its counterpart **7b**, which had an IC_50_ of 0.46 μM. This increase in activity may be due to the isosteric replacement of the benzene ring by the pyridine of the phenylamino fragment attached to the C-2 of the purine. This result indicates that the modification suggested by the 3D-QSAR was accurate.

On the other hand, in general terms, it can be appreciated that, of these seven new purine derivatives, except for **7b**, the most effective were typically those with a single substitution at the *meta*-position of the phenylamino group attached to the C-6 position of the purine. Likewise, to evaluate the effect of the proposed modifications in detail, it is necessary to compare each new purine with its respective counterpart in Figure 1. In the case of **7b**, replacement of the methyl group by the hydroxyethyl group in the piperazine moiety reduced its potency against Bcr-Abl (IC_50_ = 0.46 μM) compared to its analogue **X** (IC_50_ = 0.070 μM). In addition, the change in F by CN in C-6 linked to the phenylamino fragment of the purine was not beneficial either, as evidenced by the comparison between **7b** and **VII**. In the case of **VIII** and the new compounds **7e**, **7f**, and **7g**, it was observed that it was not better to change the disubstitutions of the two fluorine atoms to other substituents, which would indicate that a significant increase in the size of the substituents was not favourable for the activity in Bcr-Abl. Therefore, it is necessary to revise the depth proposed by the 3D-QSAR in this region. This was confirmed by comparing **VII** with **7d**, where it was evident that the exchange of a fluorine atom for a bromine atom decreased the compound’s inhibition.

Therefore, as can be seen in the above analysis, compounds **7a**–**g** presented a varied inhibitory activity and, in some cases, were adjusted in accordance with what was predicted by the 3D-QSAR models, according to the low value of the residual, as was done for **7a** (Table 3). This could be explained by the fact that the substitutions did not favour the interactions of these ligands in the Bcr-Abl binding pocket. This point will be addressed in subsequent molecular docking studies.

### 2.4. In Silico Studies for Validation Design of New Purines

**Figure 6:**
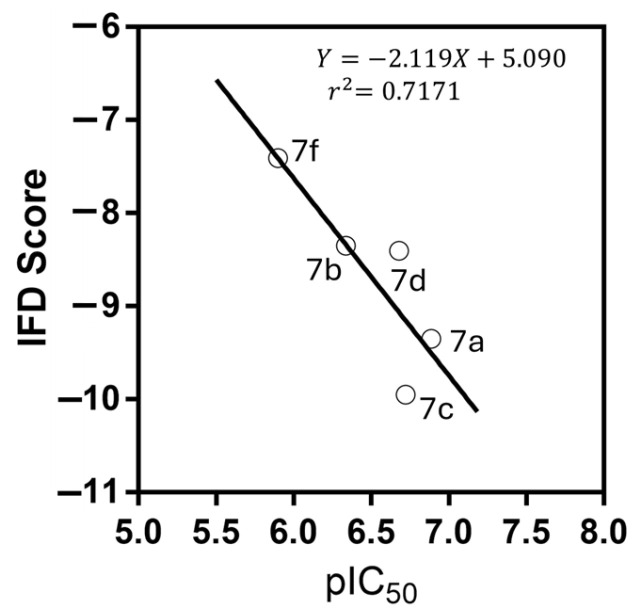
Correlation between experimental pIC_50_ and IFD score (XP GScore, kcal/mol). The equation for the line is shown along with r^2^ for the linear regression. Compounds **7e** and **7g** did not exhibit this trend.

To explain the different potencies of the designed purines as Bcr-Abl inhibitors and the different values of the experimental and predicted pIC_50_ (Table 3), docking studies were performed. The molecular docking protocol was initially validated through self-docking of the co-crystallized ligand for purvalanol B-Bcr-Abl (PDB ID: 6BL8) [31]. These purines were localised to the ATP-binding site (Figure S1), and their interaction patterns and binding energies were analysed systematically. As shown in Table 4, these purines exhibited distinct scoring values due to their structural diversity. In general, a clear trend can be observed among the pIC_50_ values and IFD scores, as shown in Figure 6. A linear relationship was observed for most of the designed compounds: **7a**, **7b**, **7c**, **7d**, and **7f**. Only compounds **7e** and **7 g** deviated from this correlation, which is an interesting result because both compounds showed the highest predicted pIC_50_ values (Table 4) and the lowest potency on Bcr-Abl.

**Figure 7:**
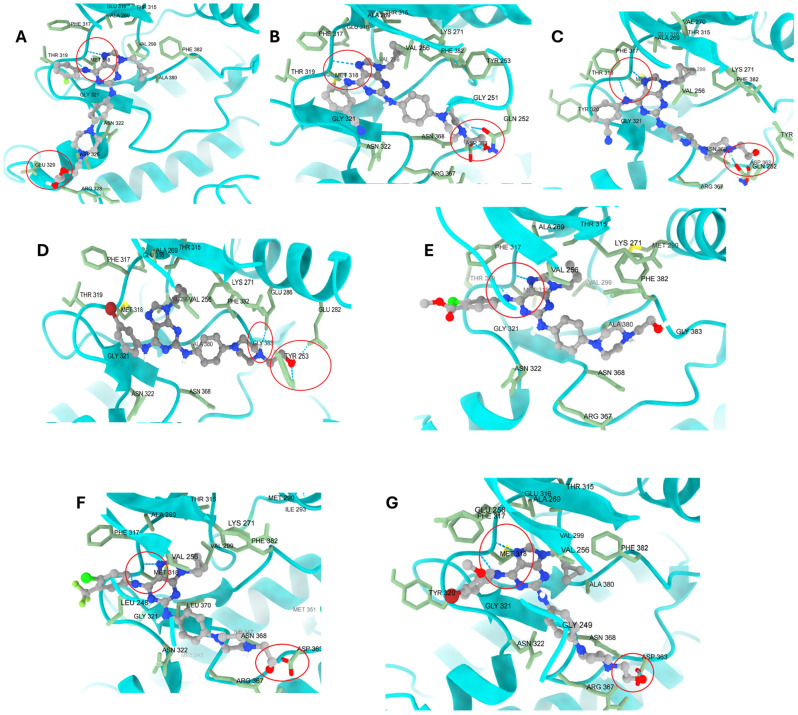
Visualization of all poses obtained from molecular docking between Bcr-Abl and **7a**–**g**. (**A**) for **7a**; (**B**) for **7b**; (**C**) for **7c**; (**D**) for **7d**; (**E**) for **7e**; (**F**) for **7f**; and (**G**) for **7g**. All compounds exhibited double interactions with the MET318 backbone, except for **7d**. In the hydroxyethyl fragment, it was observed that, in nearly all cases, it formed a hydrogen bond with ASP363, GLN252, and GLU329, generating an anchoring effect. The exception was **7e**, where this fragment did not interact with any other amino acid. The most important interactions are enclosed in a red circle.

Moreover, the binding interactions and orientation patterns of purine derivatives are critical to their Bcr-Abl inhibition. As previously reported [12], multiple interaction networks were observed between **7a** and **7g** within the ATP-binding pocket of this kinase (Figure 7A–G). **7b** and **7c** exhibited similar docking behaviour due to the only structural difference between them being the aminophenyl or aminopyridine fragment linked at C-2 of the purine. In contrast, **7a** displays a slightly different interaction profile than the other purines because the presence of the phenyl-piperazine-hydroxyethyl fragment is spatially displaced, which promotes the formation of a new hydrogen bond with GLU329. In **7g**, the *para*-bromo atom in the phenylamino fragment at C-6 is exposed to the solvent, as is the case with the *para*-chlorine atom in **7f**. This suggests that steric factors may limit the coupling values, despite retention of the double interaction with MET318. In addition, **7d** formed more hydrogen bonds than **7e**, which agrees with the experimental differences in the IC_50_ values for Bcr-Abl. This behaviour was attributed to the solvent exposure of the aniline fragment. The groups in **7e** were more exposed to the solvent than those in **7d**, as the bromine group in **7d** is non-polar while the groups in **7e** are polar. This caused the aromatic ring in **7d** to shift away from the binding site, hindering the potential formation of a π-stacking interaction with PHE317. Although **7d** could form more favourable interactions than **7e**, the polar solvent affinity effect leads the docking score to favour **7e**.

A comprehensive structural analysis corroborates the enhanced activity of our lead compounds, specifically **7a** and **7c**, in comparison to imatinib. The CoMFA and CoMSIA contour maps indicate that the incorporation of a cyclopropylmethyl group at *N*-9 in compound **7a** corresponds with sterically favorable regions, thereby augmenting the compound’s binding affinity. Additionally, the isosteric substitution of the phenyl ring with a pyridine ring at the C-2 position in compound **7c** enhances its electrostatic complementarity within the Bcr-Abl active site. Molecular docking analysis reveals that these compounds sustain critical interactions with MET318 and establish additional hydrogen bonds—such as with GLU329 in **7a** and ASP363 in **7c**—and thus stabilize their binding conformations. The increased potency of **7a** and **7c** can be attributed to their improved steric compatibility and electronic interactions, as predicted by the 3D-QSAR models and validated by docking. These findings provide a mechanistic rationale for their superior activity.

### 2.5. Cytotoxic Studies on CML Cell Lines Sensitive and Resistant to Imatinib

Initially, all compounds under investigation were evaluated for their cytotoxic effects on CML cell lines with Bcr-Abl^WT^ rearrangement and their sensitivity to imatinib, including K562 and KCL22 (CML in blast crisis), as well as on non-cancerous HEK-293T cells (Table 5). The cytotoxic effects of the 2,6,9-trisubstituted purines demonstrated variability that was contingent upon the cell type and the specific compounds being assessed. Among the cell lines, K562 was the most responsive, with all compounds showing GI_50_ values ranging from 0.30 to 4.04 μM, whereas KCL22 was the least responsive, with only five compounds exhibiting GI_50_ values below 10 μM. Compound **7c** emerged as the most effective derivative, with GI_50_ values of 0.30 and 1.54 μM for the K562 and KCL22 cell lines, respectively. Interestingly, **7c** demonstrated comparable potency to imatinib in K562 cells but was six times more potent than imatinib in KCL22 cells. It should also be noted that **7c** is more potent in K562 cells than any of the 58 previously studied purines [11,12,13]. In addition, by comparing **7c** and **7b**, it was possible to establish that the substitution of the pyridylamino fragment by the phenylamino fragment at the C-2 position of purine increased the cytotoxicity in both cells, which was consistent with enhanced Abl inhibition. Compound **7c** was 13-fold more potent than **7b** on K562 cells and approximately 8.5-fold more potent on KCL22 cells.

According to what has been previously analysed, it should be considered that, although it is important that these purines inhibit the growth of CML cell lines containing Bcr-Abl^WT^, it is equally interesting to study the response of these compounds in a cell line that has the Bcr-Abl^T315I^ mutation, KCL22-B8. This is important because this mutation is responsible for resistance to imatinib [32] and nilotinib [33]. Therefore, the KCL22-B8 cell line provides further evidence regarding the future of these purines. Looking at the results in Table 5, there was a lower sensitivity to the synthesized compounds compared to the cell lines without the mutation. However, compounds **7f**, **7e**, and **7g** presented GI_50_ values of 13.8, 16.56, and 15.43 μM, respectively, which are lower values than that presented by imatinib. These results suggested that **7f**, **7e**, and **7g** could also inhibit Bcr-Abl^T315I^.

Conversely, it is important to examine the results obtained from the HEK293T cell line, a non-neoplastic cell line, as it serves as a control in cell cytotoxicity studies, facilitating the comparison of its response with that of cancer cells. In this context, it is expected that the GI_50_ values in the HEK-293T cells will be elevated, which would indicate a lower sensitivity to the compound and, therefore, lower toxicity in healthy cells. In this study, compound **7c** showed the highest value (GI_50_ > 25 μM), followed by **7b** with a GI_50_ of 14.75 μM and **7a** with a GI_50_ of 6.85 μM. These results suggest that, among the compounds tested, **7c** was the least cytotoxic to non-tumour cells. In cytotoxicity assays, the selectivity index (SI) is an important measure that helps to evaluate the specificity of a compound to preferentially affect tumour cells versus non-tumour cells. The SI was calculated as the ratio of the GI_50_ value in a non-tumour cell line (such as HEK-293T) to the value in a cancer cell line (K562, KCL22, or KCL22-B8). The SI values of the studied purines are listed in Table 6. Based on these values, compound **7c** stands out for its high SI towards the K562 cell line (SI value = 83), which is higher than that of imatinib (SI value = 43). Other compounds showed moderately lower SI values than imatinib (5.65–1.1). For KCL22, compound **7c** again stood out, with an SI value of 16, which was higher than that of imatinib (SI value = 1.1). For **7a** and **7d**, the SI values were 2.9 and 2.7. On the other hand, in the KCL22-B8 cell line, the SI values were lower than those of other cell lines and, for almost all derivatives, those for purine were lower than those for imatinib, except for **7c**, which had an SI value = 0.8.

Finally, it should be noted that, although these newly designed purines did not show higher potency on Bcr-Abl than previous compounds, it is interesting to consider that some purines are capable of inhibiting growth in CML cells that are sensitive and resistant to imatinib and have better SI values than imatinib, such as **7c** and **7a** (for K562 and KCL22) and **7b** (for KCL22-B8).

### 2.6. In Silico Studies for Bcr-Abl^T15I^

To elucidate the antiproliferative effects of these purines on KCL22 and KCL22-B8 cells, we posited that they were connected to the presence of Bcr-Abl in either the wild-type or T315I mutant states, and then determined the degree of inhibition by these purines. To verify this hypothesis, we first conducted molecular docking studies on Bcr-Abl^T315I^ (PDB ID: 4TWP [34]) for **7e** and **7f**, the most active compounds in KCL22-B8 cells. The binding affinity energy for **7e** was −10.186 kcal/mol and for **7f** it was −10.457 kcal/mol; these energies were consistent with the GI_50_ values (15.43 for **7e** and 13.80 for **7f** μM). This result is interesting because both compounds were less active in Bcr-Abl^WT^ inhibition, and it could be hypothesized that they are more active in Bcr-Abl^T15I^.

**Figure 8:**
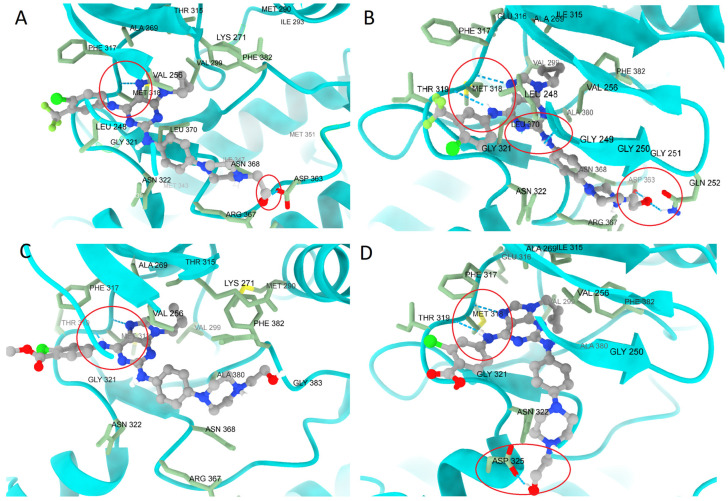
Comparison of the poses of (**A**) **7f** in Bcr-Abl^WT^, (**B**) Bcr-Abl^T315I^, (**C**) **7e** in Bcr-Abl^WT^, and (**D**) Bcr-Abl^T315I^. In both cases, the ligands remained largely unaffected by the T315I mutation, which suggests stable docking in the presence of this mutation. Additionally, each ligand gains a new hydrogen bond: **7f** interacts with GLN252, LEU248, and ASP353, while **7e** interacts with ASP325. The most important interactions are enclosed in a red circle.

Figure 8A,C depict **7f** and **7e** in Bcr-Abl^WT^, while Figure 8B,D show their docking in Bcr-Abl^T315I^. In both cases, interactions with MET318 were retained, despite the mutation. Both ligands also establish new interactions with hydroxyethyl fragments. The pose of **7e** shifts compared to its position in Bcr-Abl^WT^ due to the rotational flexibility of the ligand fragment and its polar end, which anchors with polar residues such as ASP325.

Molecular dynamics (MD) studies were conducted to investigate, in depth, the fact that **7e** and **7f** are better inhibitors of Bcr-Abl^T315I^ than Bcr-Abl^WT^. With this goal, the stability of both ligands in complexes with WT and mutated kinases were evaluated, and imatinib was also included using NAMD 3.0.1 under the conditions described in the Methods section [35]. To assess the structural stability of each system, the root-mean-square deviation (RMSD) values for the ligand atoms were calculated over 200 ns production trajectories. These analyses provide insights into global conformational changes and the extent of equilibration over time.

Figure S2 shows the RMSD profiles of the ligands in their respective complexes over the 200 ns MD simulations. The results indicated that the ligand stability varies depending on the protein state, with distinct RMSD trends being observed for imatinib, **7f**, and **7e** in both Bcr-Abl^WT^ and Bcr-Abl^T315I^. The RMSD fluctuations ranged from 1.5 to 3 Å for these compounds. The RMSD values of the proteins in their respective complexes are shown in Figure S3. Fluctuations occurred in the range of 2–5 Å, with the largest fluctuation being observed for the protein in the imatinib–Bcr-Abl^WT^ complex. The smallest fluctuation was found in the **7e** complex, both in the WT and mutated forms.

Additionally, MMGBSA calculations were performed throughout the production trajectory to estimate the ligand-binding free energies using the MolAICal v.1.3 software [36]. This approach enabled a comparative assessment of how the T315I mutation affects ligand affinity, revealing differences in the overall binding energy contributions and interaction patterns within the active site. From Table 6, we can observe that imatinib exhibits a better free energy in the WT form (−38.08 kcal/mol) than in the mutated form (−28.18 kcal/mol), which is in agreement with its IC_50_ values. In contrast, the **7e** and **7f** showed higher binding energies in the mutant form (−33.52 and −30.06 kcal/mol, respectively), which is related to their GI_50_ values on KCLL22-B8 cells.

### 2.7. Calculated Physicochemical Properties and ADME Parameters

**Figure 9:**
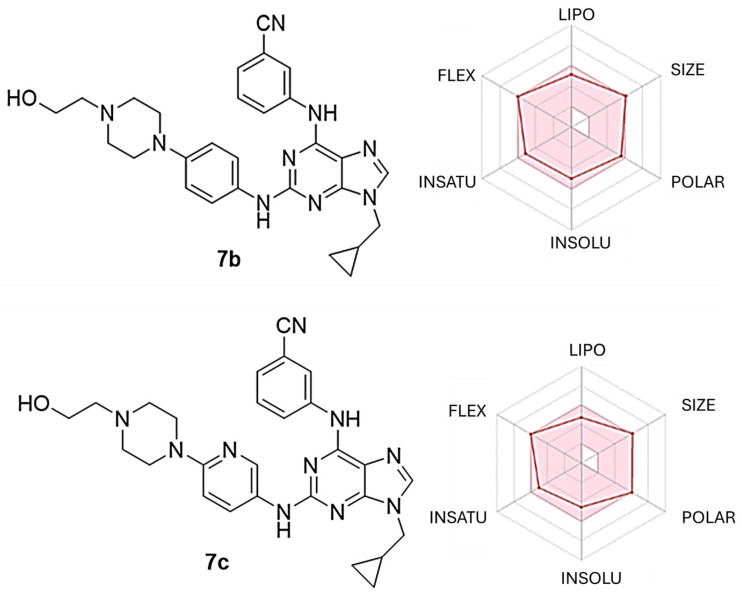
Radar plot illustrating the predicted bioavailability of compounds **7b** and **7c**. The pink region represents the optimal value range for each property associated with oral bioavailability, while the predicted properties are shown by red lines.

In the drug discovery and development process, it is essential to consider both pharmacological characteristics and pharmacokinetic profiles. Thus, predicting or determining these pharmacokinetic properties—encompassing administration, distribution, metabolism, and excretion (ADME)—is crucial for optimizing a bioactive compound until it qualifies for preclinical studies [37]. The SwissADME online platform (http://www.swissadme.ch/index.php, accessed on 1 March 2025) was utilized to evaluate the physicochemical properties of purine derivatives **7a**–**g** in accordance with Lipinski’s rules. As indicated in Table 7, among the seven purine derivatives that we analysed, only **7b** closely meets all criteria for favourable permeability and bioavailability, based on its molecular weight (MW), hydrogen bond donor (HBD), hydrogen bond acceptor (HBA), and cLogP values [38]. Furthermore, following Veber’s rules, compounds **7b–d** and **7f–g** possess a topological polar surface area (TPSA) and a number of rotatable bonds (NRB) that are below the thresholds of 140 Å^2^ and ≤10 NRB, respectively (Table 7) [39]. These metrics suggest that **7b** and **7c** are likely to penetrate cell membranes effectively and exhibit good oral absorption, in line with the Lipinski and Veber rules. Moreover, all purine derivatives exhibit high oral gastrointestinal (GI) absorption and do not penetrate the blood–brain barrier (BBB), which is favourable for minimizing potential central nervous system side effects. All compounds are substrates of P-glycoprotein, and only two of them—compounds **7b** and **7c**—show a high risk of hERG inhibition. Additionally, the SwissADME platform provided a bioavailability radar plot that allowed us to assess parameters such as flexibility (FLEX), lipophilicity (LIPO), solubility (INSOLU), size (SIZE), polarity (POLAR), and saturation (INSATU). If all parameters fall within the desired range (pink region), the compound is expected to demonstrate good oral absorption. Figure 9 illustrates that **7b** and **7c** fulfil these criteria.

## 3. Materials and Methods

### 3.1. 3D-QSAR Studies

#### 3.1.1. Selection of Conformers and Molecular Alignment

CoMFA and CoMSIA studies were conducted utilizing Sybyl X v.1.2 software (Tripos International, St. Louis, MS, USA). To identify the most suitable conformers for each molecule, every compound was drafted in ChemDraw v. 15.1.0 (PerkinElmer, Waltham, MA, USA) and subjected to initial geometry optimization via MM2 molecular mechanics, which was implemented in ChemBio3D v.15.1.0 software. The mol2 structures were imported into Sybyl, where MMFF94 charges were assigned to each atom. Superimposed molecules were minimized using Powell’s method [40].

#### 3.1.2. CoMFA and CoMSIA Field Calculation

To derive the CoMFA and CoMSIA descriptor fields, the aligned training set molecules were positioned within a three-dimensional cubic lattice, with a grid spacing of 2 Å in the x, y, and z dimensions to encompass the entire set. The CoMFA steric and electrostatic field energies were calculated utilizing a *sp*^3^ carbon probe atom with a Van der Waals radius of 1.52 Å and a charge of +1.0. The cutoff values for both the steric and electrostatic fields were set at 30.0 kcal/mol. For CoMSIA analysis, standard settings (probe with charge +1.0, radius 1 Å, hydrophobicity +1.0, H-bond donating +1.0, and H-bond accepting +1.0 [41]) were employed to compute five distinct fields: steric, electrostatic, hydrophobic, donor, and acceptor. The Gaussian-type distance dependence was utilized to evaluate the relative attenuation of the field position of each atom in the lattice, which resulted in a smoother sampling of the fields surrounding the molecules compared to CoMFA. An attenuation factor α of 0.3 was established as the default value.

#### 3.1.3. Internal Validation and Partial Least Squares (PLS) Analysis

PLS analysis was implemented to establish a linear correlation between the CoMFA and CoMSIA descriptors (independent variables) and the activity values (dependent variables) [42]. To identify the most effective model, cross-validation analysis was executed using the leave-one-out (LOO) method (and sample distance PLS [SAMPLS]) to generate the square of the cross-validation coefficient (*q*^2^) and optimal number of components (N). Non-cross validation was performed with a column filter value of 2.0 to expedite the analysis and diminish noise. *q*^2^, which is a measure of the internal quality of the models, was obtained using Equation (1):

(1) $$q^{2}=1-\frac{\sum {\left(y_{i}-y_{pred}\right)}^{2}}{\sum {\left(y_{i}-\bar{y}\right)}^{2}}$$

where $y_{i}$, $\bar{y}$, and $y_{pred}$ are observed, mean, and predicted activity in the training set, respectively.

#### 3.1.4. External Validation

The models were subjected to external validation criteria based on the test proposed by Golbraikh and Tropsha [24,25], which considers a QSAR model predictive if the following conditions are met:

(2) $$q^{2}>0.5$$

(3) $$r_{test}^{2}>0.6$$

(4) $$\frac{\left(r_{test}^{2}-r_{0}^{2}\right)}{r_{test}^{2}}<0.1\;\mathrm{or}\;\frac{\left(r_{test}^{2}-r_{\;0}^{\prime 2}\right)}{r_{test}^{2}}<0.1$$

(5) $$0.85\leq k\leq 1.15\;\mathrm{or}\;0.85\leq k^{\prime }\leq 1.15$$

(6) $$\left|r_{0}^{2}-{r^{\prime }}_{0}^{2}\right|<0.3$$

It has been demonstrated [32] that all aforementioned criteria are essential for a comprehensive evaluation of a QSAR model’s predictive capability. Furthermore, the external predictive power of the developed 3D-QSAR models employing the test set was analysed by accounting for $r_{m}^{2}$ metrics, as shown below [27]:

(7) $$r_{m}^{2}=r_{test}^{2}\;\left(1-\left|\sqrt{r_{test}^{2}-r_{0}^{2}}\right|\right)$$

where $r_{test}^{2}$ and $r_{0}^{2}$ are the squared correlation coefficients between the observed and predicted activities of the test set with and without the (0,0) intercept, respectively. For a significant external model validation, the value of $r_{m}^{2}$ should be greater than 0.5.

#### 3.1.5. Applicability Domain Calculation

The AD was evaluated using a straightforward standardization method reported by Roy et al. [43]. First, each descriptor “i” for each compound “k” was standardized (Sik). Each compound must have a maximum value of [Si]max(k) ≤ 3. In the case that [Si]max(k) > 3 and its minimum value [Si]min(k) < 3, then the Snew(k) parameter must be calculated and has to fulfil the condition Snew(k) = ${\bar{S}}_{k}+1.28*\sigma_{S_{k}}$, where ${\bar{S}}_{k}$ is the mean of Sik values for compound k and $\sigma_{S_{k}}$ is the standard deviation for such values. The software is freely accessible on the authors’ website: https://teqip.jdvu.ac.in/QSAR_Tools/ (accessed on 17 May 2025).

### 3.2. Chemistry

All reagents and chemicals utilized in the chemical synthesis of intermediates and final compounds were procured from Sigma-Aldrich (St. Louis, MO, USA). Intermediates previously published were synthesized according to established procedures, as referenced in the relevant literature. HPLC-grade solvents were acquired from Merck (Darmstadt, Germany) and Fisher Scientific (Waltham, MA, USA).

The melting points (m.p.) of all synthesized compounds were measured uncorrected using a Kofler Thermogerate apparatus (Reichert, Werke A.G., Wien, Vienna, Austria). The ^1^H and ^13^C nuclear magnetic resonance (NMR) spectra of the synthesis intermediates and final compounds were recorded on BRUKER AVANCE III HD-400 (400 MHz (^1^H) and 100 MHz (^13^C)) and 200 MHz [200 MHz (^1^H) and 50 MHz (^13^C)] spectrometers. The compounds were dissolved in CDCl_3_ or DMSO-*d*_6_, which tetramethylsilane (TMS) being utilized as an internal standard. The chemical shifts in the NMR spectra are expressed in parts per million (ppm) and coupling constants (*J*) are given in hertz (Hz) where applicable. The multiplicity observed in the ^1^H NMR spectra is indicated as s (singlet), d (doublet), t (triplet), and dd (doublet doublet). High-resolution mass spectra (HRMS) or mass spectra (MS) were obtained using a Q-TOF mass spectrometer (Synapt G2-Si, Waters, Milford, MA, USA) equipped with an electrospray ionization (ESI) source. Briefly, the measurement process involved injecting an acetonitrile solution of the sample directly into the ESI source using a syringe pump at a flow rate of 10 µL/min. Positive-mode molecular ions were detected on the Q-TOF mass spectrometer. Reaction monitoring and verification of the purity of the synthesized products post-column chromatography were conducted through thin-layer chromatography (TLC) using Merck GF-254 type 60 silica gel. The purity of the final compounds for biological assays was established via TLC, HRMS, and HPLC.

#### 3.2.1. General Procedure for the Synthesis of Intermediates **2a**–**2a**′

A solution of 2,6-dichloro-9*H*-purine (**1**, 1.0 g, 5.29 mmol), methylcyclopropyl bromide (0.79 g, 5.82 mmol), and K_2_CO_3_ (2.19 g, 15.87 mmol) in DMF (10 mL) was prepared, and the mixture was stirred at room temperature for 12 h. The mixture was then filtered and evaporated. The residue was purified by silica gel chromatography using a polar mobile phase (EtOAc/hexane, 1:1) to yield products **2a** and **2a**′.

2,6-Dichloro-9-(cyclopropylmethyl)-9*H*-purine (**2a**). White solid, yield 60%. The analytical data corresponded to the literature [13].

2,6-Dichloro-9-(cyclopropylmethyl)-7*H*-purine (**2a**′). White solid, yield 18%, and analytical data corresponded to the literature [13].

#### 3.2.2. General Procedure for the Synthesis of Intermediates **3a**–**f**

A solution of **2a** (200 mg, 0.83 mmol), the corresponding aniline (0.90 mmol), and DIPEA (0.3 mL, 1.65 mmol) in *n*-butanol (5 mL) was prepared and stirred at 110 °C for 12 h. To halt the reaction, ice water was added, and the mixture was washed twice with water, which resulted in solid formation. If two phases were noted, extraction was performed with EtOAc (3 × 15 mL), and the organic layer was dried with anhydrous Na_2_SO_4_ before concentration. The residue was purified via silica gel chromatography using a polar mobile phase (AcOEt/hexane 60:40) to acquire products **3a**–**f**.

2-Chloro-9-(cyclopropylmethyl)-*N*-(3-fluorophenyl)-9*H*-purin-6-amine (**3a**): White solid, yield 85%. The analytical data corresponded to the literature [13].

3-((2-Chloro-9-(cyclopropylmethyl)-9*H*-purin-6-yl)amino)benzonitrile (**3b**): White solid, yield 76%. The analytical data corresponded to the literature [12].

*N*-(3-bromophenyl)-2-chloro-9-(cyclopropylmethyl)-9*H*-purin-6-amine (**3c**): Brown solid, yield 54%, m.p. 127.3–129.5 °C. ^1^H NMR (400 MHz, chloroform-*d*) δ 8.17 (s, 1H, NH), 7.96 (s, 1H, CH), 7.90 (s, 1H, CH), 7.73 (m, 1H, CH), 7.22 (d, *J* = 4.7 Hz, 2H, 2 CH), 4.02 (d, *J* = 7.3 Hz, 2H, CH_2_), 1.30 (m, 1H, CH), 0.69 (t, *J* = 6.9 Hz, 2H, CH_2_), 0.45 (t, *J* = 5.2 Hz, 2H, CH_2_). ^13^C NMR (101 MHz, chloroform-*d*) δ 153.91, 152.02, 150.95, 140.86, 139.51, 130.37, 126.79, 123.03, 122.60, 119.22, 118.75, 48.74, 11.03, 4.40 (2C).

Methyl-2-chloro-4-((2-chloro-9-(cyclopropylmethyl)-9*H*-purin-6-yl)amino)benzoate (**3d**): white solid, yield 30%, m.p. 127.3–131.5 °C. ^1^H NMR (200 MHz, DMSO-*d*_6_) δ 10.74 (s, 1H, NH), 8.46 (s, 1H, CH), 8.25 (d, *J* = 2.0 Hz, 1H, CH), 7.97 (dd, *J* = 8.7, 2.1 Hz, 1H, CH), 7.86 (d, *J* = 8.7 Hz, 1H, CH), 4.04 (d, *J* = 7.2 Hz, 2H, CH_2_), 3.83 (s, 3H, CH_3_), 1.46–1.14 (m, 1H, CH), 0.64–0.36 (m, 4H, 2 CH_2_). ^13^C NMR (101 MHz, DMSO-*d*_6_) δ 165.28, 152.29, 152.11, 151.79, 143.86, 143.48, 133.38, 132.54, 123.18, 121.99, 119.76, 118.82, 52.68, 48.27, 11.60, 4.30 (2C).

2-Chloro-*N*-(4-chloro-3-(trifluoromethyl)phenyl)-9-(cyclopropylmethyl)-9*H*-purin-6-amine (**3e**): white solid, yield 60%, m.p. 103.5–105.2 °C. ^1^H NMR (400 MHz, chloroform-*d*) δ 8.70 (s, 1H, NH), 8.10 (d, *J* = 2.7 Hz, 1H, CH), 8.04–7.96 (m, 1H, CH), 7.91 (s, 1H, CH), 7.45 (d, *J* = 8.7 Hz, 1H, CH), 4.01 (d, *J* = 7.4 Hz, 2H, CH_2_), 1.29 (dq, *J* = 13.8, 6.9, 5.9 Hz, 1H, CH), 0.67 (m, 2H, CH_2_), 0.44 (m, 2H, CH_2_). ^13^C NMR (101 MHz, chloroform-*d*) δ 153.89, 151.80, 151.06, 140.88, 137.39, 132.00 (2C), 128.72 (d, ^2^*J_C-F_* = 31.3 Hz), 126.06–119.06 (dd, ^1^*J_C-F_* = 216.6 Hz) 123.86, 118.93 (q, ^3^*J_C-F_* = 5.7 Hz) 48.82, 10.99, 4.39 (2C).

*N*-(4-bromo-3-methoxyphenyl)-2-chloro-9-(cyclopropylmethyl)-9*H*-purin-6-amine (**3f**): Yellow solid, yield 83%, m.p. 115.6–117.2 °C. ^1^H NMR (400 MHz, chloroform-*d*) δ 8.20 (s, 1H, NH), 7.94–7.86 (m, 2H, 2 CH), 7.45 (d, *J* = 8.5 Hz, 1H, CH), 7.01 (dd, *J* = 8.6, 2.4 Hz, 1H, CH), 4.04 (d, *J* = 7.2 Hz, 2H, CH_2_), 3.96 (s, 3H, CH_3_), 1.32 (tdd, *J* = 10.7, 9.5, 7.9, 4.8 Hz, 1H, CH), 0.74–0.65 (m, 2H, CH_2_), 0.45 (dd, *J* = 5.1 Hz, 2H, CH_2_). ^13^C NMR (101 MHz, chloroform-*d*) δ 156.13, 153.77, 151.94, 150.82, 140.67, 138.88, 132.99, 119.17, 112.81, 105.37, 104.51, 56.20, 48.74, 11.03, 4.39 (2C).

#### 3.2.3. General Procedures for the Synthesis of Intermediates **5a**–**c**

A solution of **4a** or **4b** (7.0 mmol), 2-(2-(Piperazin-1-yl)ethoxy)ethan-1-ol or 2-hydroxyethyl piperazine (7.1 mmol), and K_2_CO_3_ (2.9 g, 21.1 mmol) in DMF (10 mL) was prepared and stirred at room temperature for 12 h. The reaction mixture was filtered and evaporated. The solid was subsequently recrystallized in MeOH to yield products **5a**–**c**.

2-(2-(4-(4-Nitrophenyl)piperazin-1-yl)ethoxy)ethan-1-ol (**5a**): Orange solid, yield 98%, m.p. 84.5 °C. ^1^H NMR (200 MHz, chloroform-*d*) 8.19–8.02 (m, 2H, 2 CH), 6.90–6.75 (m, 2H, 2 CH), 3.71 (m, 4H, 2 CH_2_), 3.63 (m, 2H, 2 CH_2_), 3.53–3.38 (m, 4H, 2 CH_2_), 2.67 (q, *J* = 5.1 Hz, 6H, 3 CH_2_). ^13^C NMR (50 MHz, chloroform-*d*) δ 154.77, 138.49, 125.88 (2C), 112.70 (2C), 72.40, 67.58, 61.85, 57.76, 52.74 (2C), 46.71 (2C).

2-(4-(4-Nitrophenyl)piperazin-1-yl)ethan-1-ol, (**5b**): Yellow solid, yield 97%. The analytical data corresponded to the literature [11].

2-(4-(5-Nitropyridin-2-yl)piperazin-1-yl)ethan-1-ol (**5c**): Yellow solid, yield 98%, m.p. 112.0–115.1 °C. 1H NMR (200 MHz, chloroform-*d*) δ 9.01 (d, *J* = 2.8 Hz, 1H, CH), 8.19 (dd, *J* = 9.5, 2.8 Hz, 1H, CH), 6.57 (d, *J* = 9.5 Hz, 1H, CH), 4.11 (m, 1H, OH), 3.85–3.76 (m, 4H, 2 CH_2_), 3.69 (t, *J* = 5.3 Hz, 2H, CH_2_), 2.63 (dt, *J* = 5.0, 2.5 Hz, 6H, 3 CH_2_). ^13^C NMR (50 MHz, chloroform-*d*) δ 160.32, 146.40, 135.05, 132.99, 104.54, 59.38, 57.91, 52.53 (2C), 44.87 (2C).

#### 3.2.4. General Procedures for the Synthesis Compounds **6a**–**c**

A solution of **5a**–**c** (500 mg, 1.69 mmol) and 10% Pd/C (34 mg) in ethanol (10 mL) was prepared under a hydrogen atmosphere. After 12 h, the reaction mixture was filtered through celite and concentrated to yield **6a**–**c**.

2-(2-(4-(4-Aminophenyl)piperazin-1-yl)ethoxy)ethan-1-ol (**6a**): Purple solid, yield 100%, m.p. 126.3–127.8 °C. ^1^H NMR (400 MHz, chloroform-*d*) δ 6.75 (d, *J* = 8.2 Hz, 2H, 2 CH), 6.58 (d, *J* = 8.2 Hz, 2H, 2 CH), 3.67–3.62 (m, 4H, 2 CH_2_), 3.58–3.54 (m, 2H, CH_2_), 3.06–2.99 (m, 4H, 2 CH_2_), 2.67–2.61 (m, 4H, 2 CH_2_), 2.59 (t, *J* = 5.5 Hz, 2H, CH_2_). ^13^C NMR (101 MHz, chloroform-*d*) δ 144.35, 140.24, 118.71 (2C), 116.20 (2C), 72.43, 67.62, 61.93, 57.91, 53.51 (2C), 50.66 (2C).

2-(4-(4-aminophenyl)piperazin-1-yl)ethan-1-ol (**6b**): Purple solid, yield 98%. The analytical data corresponded to literature [11].

2-(4-(4-aminophenyl)piperazin-1-yl)ethan-1-ol (**6c**): Purple solid, yield 98%, m.p. 129.3–133.5 °C. ^1^H NMR (200 MHz, chloroform-*d*) δ 7.79 (d, *J* = 2.9 Hz, 1H, CH), 6.98 (dd, *J* = 8.8, 3.0 Hz, 1H, CH), 6.57 (d, *J* = 8.8 Hz, 1H, CH), 3.66 (t, *J* = 5.4 Hz, 2H, CH_2_), 3.45–3.31 (m, 4H, 2 CH_2_), 3.08 (s, 2H, NH_2_ and OH), 2.61 (dt, *J* = 8.9, 5.2 Hz, 6H, 3 CH_2_). ^13^C NMR (50 MHz, chloroform-*d*) δ 154.40, 135.28, 134.72, 126.07, 108.45, 59.42, 57.79, 52.79 (2C), 46.77 (2C).

#### 3.2.5. General Procedures for the Synthesis of Final Compounds for Biological Assays **7a**–**g**

A solution of **3a**–**f** (200 mg, 0.49 mmol) and **6a**–**c** (132 mg, 0.59 mmol) in dioxane (5 mL) was stirred with Pd(OAc)_2_ (11 mg, 0.05 mmol, 0.1 eq), Xantphos (86 mg, 0.14 mmol, 0.3 eq), and 2 M K_2_CO_3_ (aq) (0.75 mL, 0.15 mmol, and 3 eq) at 170 °C for 10 min in a microwave reactor. After cooling to room temperature, the mixture was filtered through celite, and the filtrate was diluted with dichloromethane and concentrated to dryness. The residue was purified through silica gel chromatography utilizing a polar mobile phase (methanol/dichloromethane, 5:95, 10:90) to achieve the final product. The R_f_ values of each compound were determined using a 5:95 mobile phase. All compounds were >98% pure as determined by HPLC analysis (refer to Supporting Information).

2-(2-(4-(4-((9-(Cyclopropylmethyl)-6-((3-fluorophenyl)amino)-9*H*-purin-2-yl)amino)phenyl)piperazin-1-yl)ethoxy)ethan-1-ol (**7a**): Red solid, yield 52%, m.p. 60.3–61.8 °C, R_f_ = 0.4. ^1^H NMR (400 MHz, chloroform-*d*) δ 8.11 (s, 1H, NH), 7.89 (d, *J* = 11.5 Hz, 1H, CH), 7.69 (s, 1H, CH), 7.53 (d, *J* = 8.4 Hz, 2H, 2 CH), 7.30–7.17 (m, 2H, 2 CH), 7.01 (s, 1H, NH), 6.93 (d, *J* = 8.4 Hz, 2H, 2 CH), 6.73 (t, *J* = 8.3 Hz, 1H, CH), 3.93 (d, *J* = 7.2 Hz, 2H, CH_2_), 3.77–3.67 (m, 4H, 2 CH_2_), 3.64 (t, *J* = 4.3 Hz, 2H, CH_2_), 3.20 (t, *J* = 4.8 Hz, 4H, 2 CH_2_), 2.71 (t, *J* = 5.0 Hz, 4H, 2 CH_2_), 2.66 (t, *J* = 5.6 Hz, 2H, CH_2_), 1.33–1.24 (m, 1H, CH), 0.67 (d, *J* = 7.9 Hz, 2H, CH_2_), 0.45 (d, *J* = 5.1 Hz, 2H, CH_2_). ^13^C NMR (101 MHz, chloroform-*d*) δ 163.06 (d, ^1^*J_C-F_* = 243.6 Hz), 156.82, 151.84, 151.41, 146.86, 140.85 (d, ^3^*J_C-F_* = 11.0 Hz), 137.92, 133.07, 129.73 (d, *^4^J_C-F_* = 9.7 Hz), 121.24 (2C), 117.15 (2C), 115.15 (d, ^3^*J_C-F_* = 13.1 Hz), 109.28 (d, ^2^*J_C-F_* = 21.6 Hz), 107.31 (d, ^2^*J_C-F_* = 26.8 Hz), 72.48, 67.64, 61.94, 57.93, 53.42 (2C), 49.76 (2C), 48.15, 11.05, 4.30 (2C). ESI/MS for (C_29_H_35_FN_8_O [M + H]^+^): Calcd: 547.3256. Found: 547.2947.

3-((9-(Cyclopropylmethyl)-2-((4-(4-(2-hydroxyethyl)piperazin-1-yl)phenyl)amino)-9*H*-purin-6-yl)amino)benzonitrile (**7b**): Brown solid, yield 10%, m.p. 95.2–99.7 °C, R_f_ = 0.3. ^1^H NMR (400 MHz, chloroform-*d*) δ 8.45 (s, 1H, NH), 8.37 (s, 1H, NH), 7.73 (d, *J* = 6.6 Hz, 2H, 2 CH), 7.53 (d, *J* = 8.4 Hz, 2H, 2 CH), 7.32 (t, *J* = 7.8 Hz, 1H, CH), 7.28 (d, *J* = 3.1 Hz, 1H, CH), 7.15 (s, 1H, CH), 6.97 (d, *J* = 8.5 Hz, 2H, 2 CH), 3.94 (d, *J* = 7.2 Hz, 2H, CH_2_), 3.69 (t, *J* = 5.4 Hz, 2H, CH_2_), 3.19 (t, *J* = 4.8 Hz, 4H, 2 CH_2_), 3.06 (s, 1H, OH), 2.69 (t, *J* = 4.9 Hz, 4H, 2 CH_2_), 2.62 (t, *J* = 5.4 Hz, 2H, CH_2_), 1.31 (td, *J* = 8.1, 4.2 Hz, 1H, CH), 0.68 (d, *J* = 7.5 Hz, 2H, CH_2_), 0.46 (d, *J* = 5.1 Hz, 2H, CH_2_). ^13^C NMR (101 MHz, chloroform-*d*) δ 156.74, 151.60, 146.97, 140.21, 138.21, 132.76, 129.48, 126.02, 123.76, 122.67, 121.15 (2C), 119.06, 116.99 (3C), 112.66, 59.51, 57.90, 53.03 (2C), 49.90 (2C), 48.20, 30.92, 11.00, 4.32 (2C). ESI/MS for (C_28_H_31_N_9_O [M + H]^+^): Calcd: 510.2724. Found: 510.2725

3-((9-(Cyclopropylmethyl)-2-((6-(4-(2-hydroxyethyl)piperazin-1-yl)pyridin-3-yl)amino)-9*H*-purin-6-yl)amino)benzonitrile (**7c**): Yellow solid, yield 31%, m.p. 139.4–142.3 °C, R_f_ = 0.3. ^1^H NMR (400 MHz, DMSO-*d*_6_) δ 9.95 (s, 1H, NH), 9.03 (s, 1H, NH), 8.45 (d, *J* = 5.1 Hz, 2H, 2 CH), 8.28 (d, *J* = 8.2 Hz, 1H, CH), 8.07 (s, 1H, CH), 7.96 (dd, *J* = 9.0, 2.8 Hz, 1H, CH), 7.46 (dt, *J* = 16.4, 7.6 Hz, 2H, 2 CH), 6.83 (d, *J* = 9.1 Hz, 1H, CH), 4.45 (s, 1H, OH), 3.96 (d, *J* = 7.2 Hz, 2H, CH_2_), 3.55 (t, *J* = 6.2 Hz, 2H, CH_2_), 3.40 (q, *J* = 8.7, 7.0 Hz, 8H, 4 CH_2_), 2.44 (t, *J* = 6.2 Hz, 2H, CH_2_), 1.32 (ddt, *J* = 10.7, 7.6, 3.9 Hz, 1H, CH), 0.56 (q, *J* = 5.4, 4.7 Hz, 2H, CH_2_), 0.47 (t, *J* = 5.0 Hz, 2H, CH_2_). ^13^C NMR (101 MHz, DMSO-*d*_6_) δ 156.75, 155.39, 152.05, 152.01, 141.48, 139.71, 130.75, 130.13, 129.28, 125.90, 125.21, 123.07, 119.56, 115.20, 111.84, 107.28 (2C), 60.86, 59.03, 53.54 (2C), 47.59, 45.89 (2C), 11.77, 4.26 (2C). ESI/MS for (C_27_H_30_N_10_O [M + H]^+^): Calcd: 511.2677. Found: 511.2677.

2-(4-(4-((6-((3-Bromophenyl)amino)-9-(cyclopropylmethyl)-9*H*-purin-2-yl)amino)phenyl)piperazin-1-yl)ethan-1-ol (**7d**): Yellow solid, yield 30%, m.p. 109.4–114.3 °C, R_f_ = 0.3. ^1^H NMR (200 MHz, chloroform-*d*) δ 8.07 (s, 1H, NH), 7.76 (dd, *J* = 8.3, 1.2 Hz, 2H, NH, CH), 7.68 (s, 1H, CH), 7.59–7.51 (m, 2H), 7.37–7.30 (m, 1H, CH), 7.12–7.01 (m, 2H, 2 CH), 6.96–6.86 (m, 2H, 2 CH), 3.93 (d, *J* = 7.1 Hz, 2H, CH_2_), 3.71 (d, *J* = 4.1 Hz, 2H, CH_2_), 3.16 (dd, *J* = 6.4, 3.5 Hz, 4H, 2 CH_2_), 3.02 (s, 1H, OH), 2.73–2.62 (m, 4H, 2 CH_2_), 1.38–1.26 (m, 1H, CH), 0.75–0.57 (m, 2H, CH_2_), 0.44 (dt, *J* = 6.5, 4.8 Hz, 2H, CH_2_). ^13^C NMR (50 MHz, chloroform-*d*) δ 156.79, 152.17, 151.21, 146.62, 139.16, 137.67, 133.41, 128.77 (2C), 122.97, 120.87 (2C), 120.30 (2C), 116.99 (2C), 115.14, 67.07, 59.49, 57.87, 53.03 (2C), 50.13 (2C), 48.10, 11.04, 4.27 (2C). ESI/MS for (C_27_H_31_BrN_8_O [M − Br]^−^): Calcd: 483.2842 Found: 483.2596.

Methyl 2-chloro-4-((9-(cyclopropylmethyl)-2-((4-(4-(2-hydroxyethyl)piperazin-1-yl)phenyl)amino)-9*H*-purin-6-yl)amino)benzoate (**7e**): Brown solid, yield 22%, m.p. 241.4–245.3 °C, R_f_ = 0.3. ^1^H NMR (400 MHz, chloroform-*d*) δ 8.30 (s, 1H, NH), 8.21–8.13 (m, 1H, NH), 7.98 (d, *J* = 8.5 Hz, 1H, CH), 7.85 (dd, *J* = 8.6, 2.0 Hz, 1H, CH), 7.72 (s, 1H, CH), 7.54 (t, *J* = 8.6 Hz, 2H, 2 CH), 6.99–6.92 (m, 3H, 3 CH), 3.95 (dd, *J* = 7.3, 3.5 Hz, 2H, CH_2_), 3.91 (d, *J* = 2.3 Hz, 3H, CH_3_), 3.70 (t, *J* = 5.4 Hz, 2H, CH_2_), 3.20 (q, *J* = 5.1 Hz, 4H, 2 CH_2_), 2.72 (q, *J* = 5.3 Hz, 4H, 2 CH_2_), 2.64 (td, *J* = 5.6, 3.0 Hz, 2H, CH_2_), 0.86 (dt, *J* = 19.1, 6.9 Hz, 1H, CH), 0.72–0.62 (m, 2H, CH_2_), 0.50–0.43 (m, 2H, CH_2_). ^13^C NMR (101 MHz, chloroform-*d*) δ 166.87, 151.60, 143.61, 138.15, 130.71 (2C), 123.85, 121.43 (2C), 118.81 (2C), 117.13, 116.97 (2C), 59.41, 57.82, 52.99 (2C), 50.04 (2C), 49.96, 48.21, 29.70, 11.05, 4.32 (2C). ESI/MS for (C_29_H_33_ClN_8_O_3_ [M + H]^+^): Calcd: 577.2437. Found: 577.2445.

2-(4-(4-((6-((4-Chloro-3-(trifluoromethyl)phenyl)amino)-9-(cyclopropylmethyl)-9*H*-purin-2-yl)amino)phenyl)piperazin-1-yl)ethan-1-ol (**7f**): Brown solid, yield 32%, m.p. 232.4–236.4 °C, R_f_ = 0.3. ^1^H NMR (400 MHz, DMSO-*d*_6_) δ 9.81 (s, 1H, NH), 8.70 (s, 1H, NH), 8.30 (d, *J* = 9.1 Hz, 1H, CH), 8.07 (s, 1H, CH), 7.82 (s, 1H, CH), 7.32 (t, *J* = 8.4 Hz, 3H, 3 CH), 6.62 (d, *J* = 8.5 Hz, 2H, 2 CH), 4.19 (s, 1H, OH), 3.72 (d, *J* = 7.2 Hz, 2H, CH_2_), 3.30 (d, *J* = 13.5 Hz, 2H, CH_2_), 3.10 (s, 2H, CH_2_), 2.44–2.15 (m, 8H, 4 CH_2_), 1.09 (m, 1H, CH), 0.27 (dd, *J* = 6.3 Hz, 4H, 2 CH_2_). ^13^C NMR (101 MHz, DMSO-*d*_6_) δ 156.59, 152.16, 151.86, 146.38, 140.16, 139.80, 133.73, 131.89, 127.09 (t, ^2^*J_C-F_* = 25.5 Hz), 126.48–120.09 (dd, ^1^*J_C-F_* = 187.3 Hz), 125.42 (dd, ^3^*J_C-F_* = 6.0 Hz), 122.68 (d, ^2^*J_C-F_* = 15.3 Hz), 120.81 (dt, ^3^*J_C-F_* = 7.3 Hz), 119.55 (dd, ^4^*J_C-F_* = 3.6 Hz), 116.32 (2C), 115.14, 60.75, 58.99, 53.74 (2C), 49.52 (2C), 47.58, 11.79, 4.25 (2C). ESI/MS for (C_28_H_30_ClF_3_N_8_O [M + H]^+^): Calcd: 587.2256. Found: 587.2274.

2-(4-(4-((6-((4-Bromo-3-methoxyphenyl)amino)-9-(cyclopropylmethyl)-9*H*-purin-2-yl)amino)phenyl)piperazin-1-yl)ethan-1-ol (**7g**): Brown solid, yield 30%, m.p. 79.5–83.5 °C, R_f_ = 0.3. ^1^H NMR (400 MHz, chloroform-d) δ 8.07 (s, 1H, NH), 7.70 (s, 1H, CH), 7.50 (d, *J* = 8.6 Hz, 2H, 2 CH), 7.45–7.36 (m, 2H, CH, NH), 7.21 (dd, *J* = 8.7, 2.4 Hz, 1H, CH), 6.97 (s, 1H, CH), 6.90 (d, *J* = 8.6 Hz, 2H, 2 CH), 3.94 (d, *J* = 7.1 Hz, 2H, CH_2_), 3.72 (s, 3H, CH_3_), 3.69 (t, *J* = 5.4 Hz, 2H, CH_2_), 3.16 (t, *J* = 4.8 Hz, 4H, 2 CH_2_), 2.70 (t, *J* = 5.0 Hz, 4H, 2 CH_2_), 2.63 (t, *J* = 5.4 Hz, 2H, CH_2_), 1.32 (dtd, *J* = 12.8, 7.9, 5.2 Hz, 1H, CH), 0.66 (m, 2H, CH_2_), 0.45 (d, *J* = 5.2 Hz, 2H, CH_2_). ^13^C NMR (101 MHz, chloroform-d) δ 156.76, 155.97, 151.93, 151.42, 146.93, 139.77, 137.96, 133.07, 132.89, 121.27 (2C), 116.94 (3C), 115.26, 113.43, 104.64, 59.44, 57.87, 56.15, 53.00 (2C), 50.05 (2C), 48.15, 11.07, 4.30 (2C). ESI/MS for (C_28_H_33_BrN_8_O_2_ [M + H]^+^): Calcd: 593.1983. Found: 593.1987.

### 3.3. Biological Activity

#### 3.3.1. Kinase Assays

The inhibitory activity of the synthesized compounds was assessed on an in-house produced recombinant Abl1 kinase. The assay utilized 500 µM peptide GGEAIYAAPFKK as a substrate in the presence of 1 µM ATP + [γ-33P]ATP (0.05 µCi per reaction) and the test compound in a final volume of 10 µL of reaction buffer (60 mM HEPES-NaOH, pH 7.5, 3 mM MgCl_2_, 3 mM MnCl_2_, 3 μM Na-orthovanadate, 1.2 mM DTT, 2.5 μg/50 μL PEG20.000). The reaction was terminated by adding 5 µL of 3% aq. H3PO4. Aliquots were spotted onto P-81 phosphocellulose (Whatman, Maidstone, UK), washed three times with 0.5% aq. H_3_PO_4_, and air-dried. Peptide phosphorylation was quantified through digital autoradiography (FLA-7000; Fujifilm, Tokyo, Japan). The concentration of test compounds required to reduce activity by 50% was determined from the dose–response curves and reported as the IC50 value.

#### 3.3.2. Cell Cultures

The human cell line KCL22 was sourced from the German Collection of Microorganisms and Cell Cultures, while K562 and HEK-293T cells were obtained from the European Collection of Authenticated Cell Cultures. The development of the KCL22 subclone B8 (T315I, 100%) has been previously detailed [44]. All cell lines were maintained in a humidified incubator at 37 °C and 5% CO_2_, following the manufacturer’s instructions, in the appropriate medium supplemented with 10% foetal bovine serum, penicillin (100 IU/mL), streptomycin (100 µg/mL), and glutamine (4 mM).

#### 3.3.3. Cytotoxicity Assay

Cytotoxicity was assessed utilizing resazurin, an indicator dyes that measures oxidation-reduction reactions in the mitochondria of living cells. Cells were seeded into 96-well plates and treated with the compounds for 72 h (six different doses of each compound, in triplicate). After treatment, a solution of resazurin (Merck, Prague, Czech Republic) was added for 4 h, and the fluorescence of resorufin corresponding to viable cell quantity was measured at 544 nm/590 nm (excitation/emission) using a Fluoroskan Ascent microplate reader (LabSystems, Helsinki, Finland). The GI_50_ value, representing the drug concentration lethal to 50% of the cells, was calculated from the dose–response curves using Origin 6.0.

### 3.4. Molecular Docking Studies

Energy minimization and protonation of the proposed compounds were conducted using the LigPrep tool in the Maestro Schrödinger suite, version 11.8 (Schrödinger, LLC, New York, NY, 2019). For induced molecular docking, the crystal structure of the Bcr-Abl receptor (PDB code: 6BL8) was employed alongside the designed structures. Protein optimization was accomplished using the Protein Preparation Wizard available in Maestro v.14.1 software. Water molecules were removed, and appropriate hydrogen bonds were added at a pH of 7.0 ± 0.5, ensuring correct ionization states for acidic and basic amino acid residues. The receptor was optimized utilizing the OPLS4 force field. The active site was defined by a grid with a 14 Å radius centred around the Met318 residue. Initial induced docking was performed using an energy grid-based ligand docking algorithm (Glide v7.0, Schrödinger v11.2). The next step combined rigid receptor docking and side-chain protein remodelling, and included employing search and minimization techniques with the Prime module. During the protein modelling phase, all residues within a 5 Å radius of each initially docked compound were refined using Prime. After this refinement, the compounds were re-docked into the optimized receptor structure using Glide. The optimal orientations for each docked compound were identified using Glide in standard precision (SP) mode and subsequently recalculated using the Glide score function (GScore) in extra-precision (XP) mode.

### 3.5. Molecular Dynamics

The PDB structures for wild-type Bcr-Abl (PDB ID: 6BL8) and the T315I mutant (PDB ID: 4TWP) were retrieved from the Protein Data Bank. Both structures were prepared utilizing CHARMM-GUI [45] under the CHARMM36 force field for proteins and ions, and CGenFF for ligands [46]. A simulation box measuring approximately 116 × 110 × 100 Å was established, with a cutoff of 12 Å (and switching from 10 Å), and included 0.15 M of Na^+^ and Cl^−^ ions to neutralize the system and reflect physiological ionic strength. Subsequently, a 10,000-step energy minimization was executed to eliminate unfavourable contacts, followed by 2000 steps of heating to ramp from 0 K to 298 K. Next, a 50 ns equilibration was conducted to stabilize the system and to gradually release positional restraints. The ensuing 200 ns production phase was performed under NPT conditions at 298 K and 1 atm using NAMD [35]. Throughout the simulations, the Particle Mesh Ewald (PME) function was utilized for long-range electrostatics with a tolerance of 1 × 10^−6^, while a switching function was applied from 10 Å to the 12 Å cutoff for Van der Waals and real-space electrostatic interactions. A multiple time-step scheme was used which employed a 2 fs integration step for bonded and real-space non-bonded interactions, and all hydrogen-involving bonds were constrained by SHAKE [47]. The water model used was the TIP3P [48].

## 4. Conclusions

In this study, we performed a comprehensive analysis of 58 purine derivatives with inhibitory effects on Bcr-Abl that were previously synthesized by our team to develop 3D-QSAR models for the design of novel potential treatments for CML. We constructed and validated two 3D-QSAR models, CoMFA and CoMSIA, that exhibited satisfactory statistical parameters. The inhibition of Bcr-Abl was attributed to its steric, electrostatic, and hydrophobic properties. Using these data, we designed and synthesized seven new 2,6,9-trisubstituted purines. Compounds **7a** and **7c** demonstrated the strongest inhibitory activity against Bcr-Abl, surpassing the potency of imatinib, which indicated that the modifications proposed by the 3D-QSAR models were effective. Molecular docking studies revealed that these purines occupy the same binding site on Bcr-Abl. Notably, **7c** exhibited significant cytotoxicity in two CML cell lines that are sensitive to imatinib, K562 and KCL22, indicating that it is the most potent purine developed by our group. Furthermore, based on the viability of HEK293T cells, **7c** showed greater selectivity for K562 and KCL22 cells than imatinib. However, the most intriguing findings emerged when these purines were tested on imatinib-resistant KCL22-B8 cells expressing Bcr-Abl^T315I^, in which **7e** and **7f** were more effective in inhibiting cell growth. Molecular dynamics studies elucidated the key interactions of these inhibitors at the binding sites of Bcr-Abl^WT^ and Bcr-Abl^T315I^, emphasizing the differences in potency against both kinases and validating our hypothesis concerning the significance of specific fragments of the purine ring. At last, **7b** and **7c** were anticipated to possess favourable pharmacokinetic profiles for oral administration. Our findings will aid in the development of two validated 3D-QSAR models for designing potential CML drugs based on the purine scaffold.

## Figures and Tables

**Table 1:** Summary of accepted models.

								Contribution (%)		
	N	q^2^	r^2^	SEE	F	SEP	r^2^_pred_	Steric	Electrostatic	Hydrophobic
CoMFA-SE	6	0.576	0.939	0.241	82,729	0.637	0.863	0.587	0.413	
CoMSIA-SEH	6	0.637	0.934	0.252	74,988	0.589	0.842	0.249	0.472	0.279

**Table 2:** Overview of external validation parameters for CoMFA and CoMSIA.

Condition	Parameters	Threshold Value	CoMFA	CoMSIA
**1**	$q^{2}$	>0.5	0.576	0.637
**2**	$r_{test}^{2}$	>0.6	0.863	0.842
**3a**	$r_{0}^{2}$	Close to value of $r_{test}^{2}$	0.999	0.999
**3b**	${r^{\prime }}_{0}^{2}$	Close to value of $r_{test}^{2}$	0.999	0.999
**4a**	k	0.85 < k < 1.15	1.023	1.012
**4b**	$k^{\prime }$	0.85 < $k^{\prime }$ < 1.15	0.976	0.988
**5a**	$(r^{2}-r_{0}^{2})/r^{2}$	<0.1	−0.157	−0.186
**5b**	$(r^{2}-{r^{\prime }}_{0}^{2})/r^{2}$	<0.1	−0.157	−0.186
**6**	$\left|r_{0}^{2}-{r^{\prime }}_{0}^{2}\right|$	<0.3	0.00	0.00
**7**	$r_{m}^{2}$	>0.5	0.545	0.508

q^2^ = the square of the leave-one-out (LOO) cross-validation (CV) coefficient; r^2^_test_ = the regression coefficient for the test set compounds; r^2^_0_ and k are the correlation coefficients between the experimental (x) and predicted activities (y) for the test set through the origin (0, 0) and the respective slope of regression; and r′^2^_0_ and k′ are the correlation coefficients between the predicted (y) and experimental activities (x) for the test set through the origin (0, 0) and the respective slope of regression. r^2^_m_ is defined in Equation (6).

**Table 3:** The IC_50_ values for the in vitro inhibition of Abl1 kinase by purines, along with the predicted pIC_50_ values derived from CoMFA and CoMSIA models and their corresponding residuals.

Compound	IC_50_ (µM) ^a^		CoMFA		CoMSIA	
	Abl	pIC_50_ Exp	pIC_50_ Pred	Residual	pIC_50_ Pred	Residual
**7a**	0.13 ± 0.03	6.886	7.111	−0.23	7.347	−0.46
**7b**	0.46 ± 0.15	6.337	7.845	−1.51	7.880	−1.54
**7c**	0.19 ± 0.05	6.721	7.957	−1.24	7.960	−1.24
**7d**	0.21 ± 0.01	6.678	9.881	−3.20	7.187	−0.51
**7e**	0.42 ± 0.08	6.377	7.761	−1.38	7.836	−1.46
**7f**	1.26 ± 0.65	5.899	7.906	−2.01	7.450	−1.55
**7g**	0.79 ± 0.14	6.102	7.904	−1.80	7.740	−1.64
**imatinib**	0.33 ± 0.21	-	-	-	-	-

^a^ IC_50_ values were determined from at least three independent experiments.

**Table 4:** Comparison of the inducing fit docking score in Bcr-Abl and experimental pIC_50_ values.

Compound	pIC_50_	IFD Score (kcal/mol)
**7a**	6.886	−9.348
**7b**	6.337	−8.347
**7c**	6.721	−9.950
**7d**	6.678	−8.403
**7e**	6.377	−9.903
**7f**	5.899	−7.408
**7g**	6.102	−9.762

**Table 5:** In vitro cytotoxicity is expressed as GI_50_ values of purines on CML cell lines and non-neoplastic cells (HEK-293T) along with corresponding selectivity index (SI) values.

	GI_50_ (μM) ^a^						
Compound	K562	KCL22	KCL22-B8	HEK-293T	SI ^b^	SI ^b^	SI ^b^
**7a**	1.37 ± 0.61	2.38 ± 1.12	17.37 ± 2.02	6.85 ± 0.66	5.0	2.9	0.4
**7b**	4.04 ± 1.87	13.08 ± 2.21	17.90 ± 2.71	14.75 ± 0.91	3.6	1.1	0.8
**7c**	0.30 ± 0.11	1.54 ± 1.02	>25	>25	82	16	-
**7d**	1.44 ± 0.91	2.96 ± 0.97	20.08 ± 2.35	7.87 ± 1.53	5.5	2.7	0.4
**7e**	2.19 ± 1.42	3.88 ± 1.46	15.43 ± 0.98	5.98 ± 2.78	2.7	1.7	0.4
**7f**	3.56 ± 0.52	12.42 ± 4.28	13.80 ± 3.05	3.96 ± 0.57	1.1	0.3	0.3
**7g**	2.34 ± 1.04	5.51 ± 1.08	16.56 ± 0.92	4.32 ± 1.42	1.8	0.8	0.3
**imatinib**	0.23 ± 0.01	9.26 ± 1.00	>20	>10	43	1.1	0.5

^a^ GI_50_ values were determined in at least three independent experiments using the resazurin assay, and the results are expressed as GI_50_ ± SD values. ^b^ Selectivity index (SI) value = GI_50_ for HEK-293T cells/GI_50_ on CML cells. When the GI_50_ value on cells was >25, 20, or 10 μM, it was considered 25, 20, or 10 μM, respectively.

**Table 6:** Binding free energies (ΔG) from MMGBSA calculations for Bcr-Abl^WT^ and Bcr-Abl^T315I^ complexes.

	MMGBSA			
Compound	∆G Bcr-Abl^WT^ (kcal/mol)	Error	∆G Bcr-Abl^T315I^ (kcal/mol)	Error
**7e**	−26.83	0.22	−33.52	0.11
**7f**	−26.72	0.10	−32.06	0.11
**Imatinib**	−38.08	0.14	−28.18	0.07

**Table 7:** Calculated physicochemical properties, ADME parameters, and toxicity predicted for **7a**–**g**.

Compound	MW (Da)	HBA	HBD	cLogP	TPSA (Å^2^)	NRB	GI	BBB Permeant	P-gp Substrate	hERG
Desirable Value	≤500	≤10	≤5	≤5	≤140	≤10				
**7a**	546.64	7	3	3.38	103.60	12	High	No	Yes	medium risk
**7b**	509.61	6	3	2.76	118.16	9	High	No	Yes	high risk
**7c**	510.59	7	3	2.21	131.05	9	High	No	Yes	high risk
**7d**	563.49	5	3	3.61	94.37	9	High	No	Yes	medium risk
**7e**	577.08	7	3	3.36	120.67	11	High	No	Yes	medium risk
**7f**	587.04	8	3	4.43	94.37	10	High	No	Yes	medium risk
**7g**	593.52	6	3	3.53	103.60	10	High	No	Yes	medium risk

MW: molecular weight; HBA: number of hydrogen bond acceptors; HBD: number of hydrogen bond donors; cLogP: consensus Log P value; TPSA: topological polar surface; NRB: number of rotatable bonds. GI: gastrointestinal; BBB: blood–brain barrier; P-gp: P-glycoprotein; hERG: human ether-à-go-go-related gene channel, risk of inhibition. All values predicted by SwissADME (http://www.swissadme.ch/index.php, accessed on 1 March 2025).

## Data Availability

The original contributions presented in the study are included in the article and Supplementary Materials, further inquiries can be directed to the corresponding authors.

## Supplementary Materials

The following supporting information can be downloaded at https://www.mdpi.com/article/10.3390/ph18060925/s1, Table S1. The Chemical structure of the 58 in-house purine compounds; Table S2. Statistical parameters and Field combinations for CoMFA and CoMSIA; Table S3. Experimental and predicted pIC50 and residual values for the studied compounds according to CoMFA and CoMSIA. Table S4. q^2^ and r^2^ncv values after several Y-randomization tests; ^1^H-, ^13^C-spectra, HRMS, HPLC chromatograms of selected compounds. Figure S1. Representation of the Bcr-Abl surface, providing an overview of the binding site location and the conformations adopted by the studied molecules during the docking process (PDB = 6BL8); Figure S2. Time evolution of the RMSD for Bcr-Abl complexes with different ligands. Six systems were analysed: imatinib-Bcr-Abl^WT^ (blue), imatinib-Bcr-Abl^T315I^ (red), **7f**-Bcr-Abl^WT^ (green), **7f**-Bcr-AblT^315I^ (purple), **7e**-Bcr-Abl^WT^ (orange), and **7e**-Bcr-Abl^T315I^ (brown); Figure S3. Time evolution of the RMSD for the Bcr-Abl protein in complex with different ligands. Six systems were analysed: imatinib-Bcr-AblWT (blue), imatinib-Bcr-Abl^T315I^ (red), **7f**-Bcr-Abl^WT^ (green), **7f**-Bcr-Abl^T315I^ (purple), **7e**-Bcr-Abl^WT^ (orange), and **7e**-Bcr-Abl^T315I^ (brown).
