# Supplementary material for: 3D-QSAR Design of New Bcr-Abl Inhibitors Based on Purine Scaffold and Cytotoxicity Studies on CML Cell Lines Sensitive and Resistant to Imatinib
Source: Pharmaceuticals (Basel). 2025 Jun 19;18(6):925. doi: 10.3390/ph18060925 (PMC12195648; doi:10.3390/ph18060925)
Supplement: Supplementary file 1 [file pharmaceuticals-18-00925-s001.zip › pharmaceuticals-3683015-supplementary.pdf]

Supplementary Material

3D-QSAR design of new Bcr-Abl inhibitors based on purine scaffold and cytotoxicity studies on CML cell lines sensitive and resistant to imatinib

David Cabezas, [1] Thalía Delgado, [2] Guisselle Sepúlveda, [2] Petra Krňávková, [3] Veronika Vojáčková, [3] Vladimír Kryštof, [3] Miroslav Strnad, [4] Nicolás Silva, [2] Javier Echeverria, [5] Christian Espinosa-Bustos [6], Guido Mellado,[7] Jiao Luo, [1] Jaime Mella, [1,8,*] and Cristian O. Salas [2,*]

[1] Instituto de Química, Facultad de Ciencias, Universidad de Valparaíso, 2360102, Valparaíso, Chile; david.cabezas@postgrado.uv.cl (D.C.); jiao.luo@postgrado.uv.cl (J.L.)

[2] Departamento de Química Orgánica, Facultad de Química y de Farmacia, Pontificia Universidad Católica de Chile, 702843, Santiago de Chile, Chile; tdelgado@uc.cl (T.D.); gsepulvedas@uc.cl (G.S.); nsilvas@estudiante.uc.cl (N.S.)

[3] Department of Experimental Biology, Palacký University, Slechtitelu 27, 77900 Olomouc, Czech Republic; vladimir.krystof@upol.cz

[4] Laboratory of Growth Regulators, Palacký University and Institute of Experimental Botany, The Czech Academy of Sciences, Slechtitelu 27, 77900 Olomouc, Czech Republic; miroslav.strnad@upol.cz

[5] Departamento de Ciencias del Ambiente, Facultad de Química y Biología, Universidad de Santiago de Chile, Santiago 9170022, Chile; javier.echeverriam@usach.cl

[6] Departamento de Farmacia, Facultad de Química y de Farmacia, Pontificia Universidad Católica de Chile, 702843, Santiago de Chile, Chile; ccespino@uc.cl

[7] Departamento de ingeniería informática, Facultad de Ingeniería, Universidad Católica de Temuco, 4780000, Temuco, Chile; gmellado@uct.cl

[8] Centro de Investigación Desarrollo e Innovación de Productos Bioactivos (CInBIO), Escuela de Química y Farmacia, Facultad de Farmacia, Universidad de Valparaíso, 2360102, Valparaíso, Chile

* Correspondence: jaime.mella@uv.cl (J.M.), cosalas@uc.cl (C.O.S.)

Index

Table S1. The chemical structure of the 58 in-house purine compounds..pages 2- 7

Table S2. Statistical parameters and field combinations for CoMFA and CoMSIA...................................page 8

Table S3. Experimental and predicted pIC_{50} and residual values for the studied compounds according to CoMFA and CoMSIA……………………………………………………………………………………………………page 9

Table S4. q^2 and r^2_{ncv} values after several Y-randomization tests…………………………………………page 10

^{1}H and ^{13}C NMR spectra of selected compounds………………………………………………………pages 11 - 17

Mass spectra of compounds……………………………………………………………………………………pages 18 - 21

HPLC analysis of compounds ……………………………………………………………………………… pages 22 – 25

Figure S1. Representation of the Bcr-Abl surface, providing an overview of the binding site location and the conformations adopted by the studied molecules during the docking process (PDB = 6BL8)……page 26

Figure S2. Time evolution of the RMSD for Bcr-Abl complexes with different ligands. Six systems were analysed: imatinib-Bcr-Abl^{WT} (blue), imatinib-Bcr-Abl^{T315I} (red), **7f**-Bcr-Abl^{WT} (green), **7f**-Bcr-Abl^{T315I} (purple), **7e**-Bcr-Abl^{WT} (orange), and **7e**-Bcr-Abl^{T315I} (brown)…………………………………………………………………………………page 27

Figure S3. Time evolution of the RMSD for the Bcr-Abl protein in complex with different ligands. Six systems were analysed: imatinib-Bcr-Abl^{WT} (blue), imatinib-Bcr-Abl^{T315I} (red), **7f**-Bcr-Abl^{WT} (green), **7f**-Bcr-Abl^{T315I} (purple), **7e**-Bcr-Abl^{WT} (orange), and **7e**-Bcr-Abl^{T315I} (brown)……………………………………………………………page 28

Table S1. The Chemical structure of the 58 in-house purine compounds.

N°	Structure	N°	Structure
1	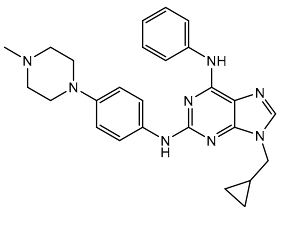	30	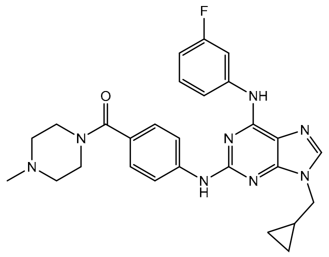
2	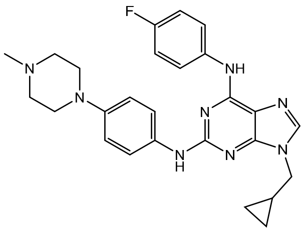	31	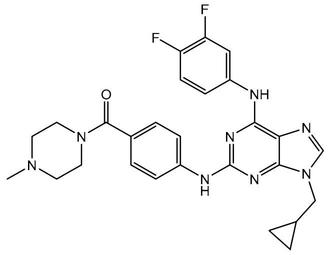

3

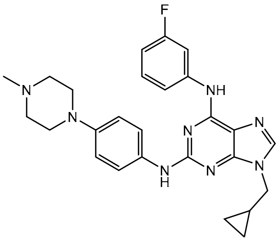

32

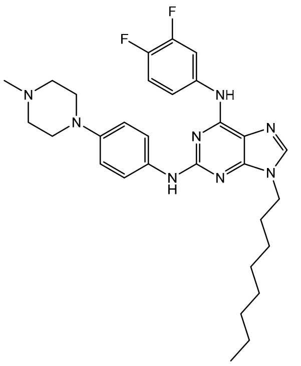

4

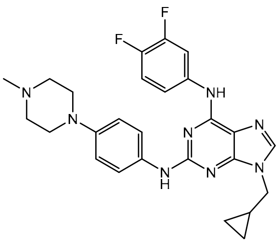

33

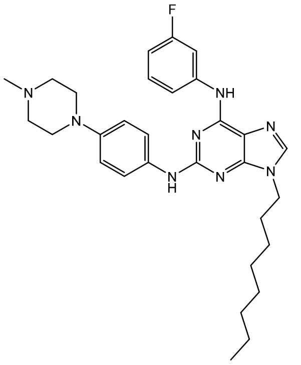

5

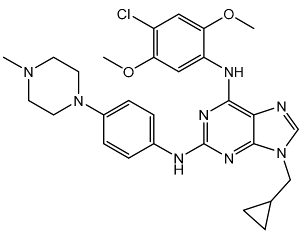

34

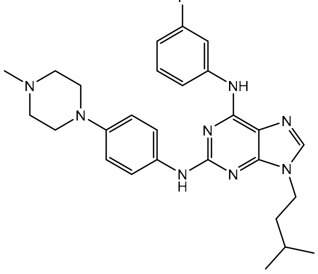

6

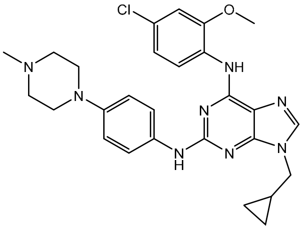

35

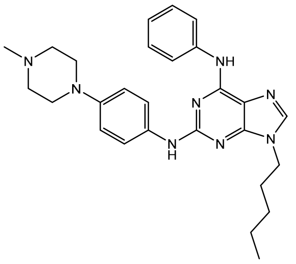

7

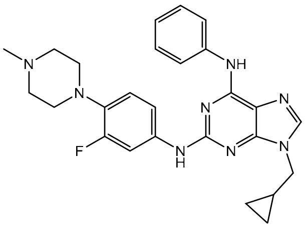

36

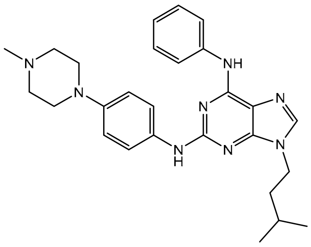

8

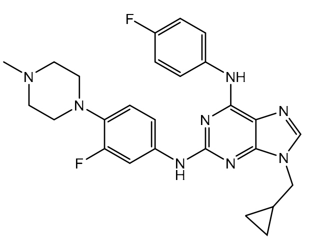

9

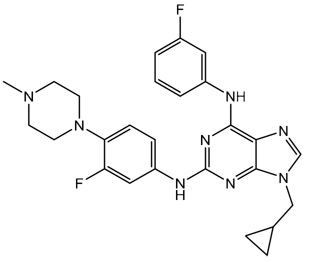

10

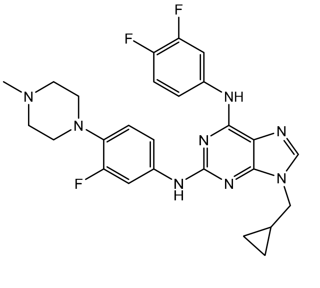

11

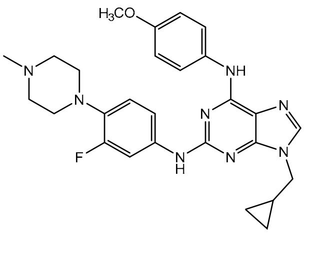

12

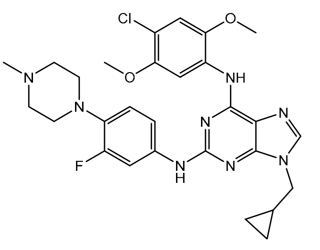

37

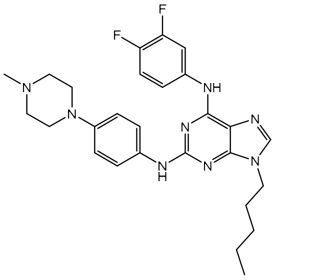

38

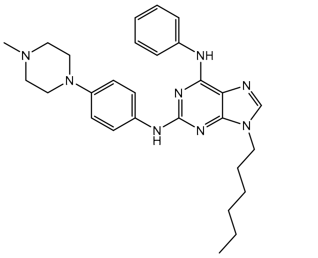

39

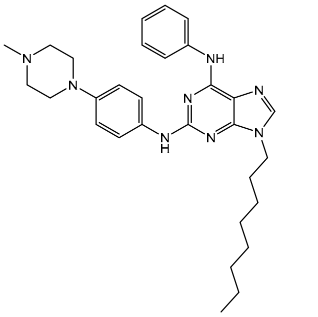

40

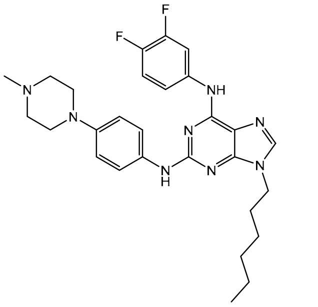

41

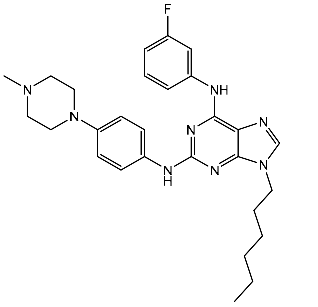

13

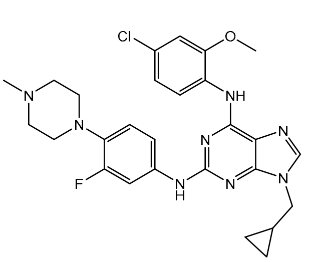

14

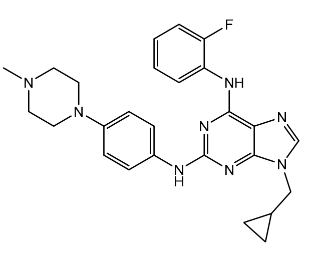

15

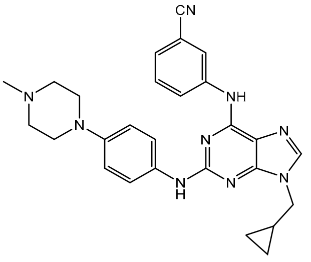

16

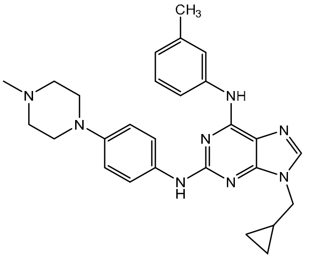

17

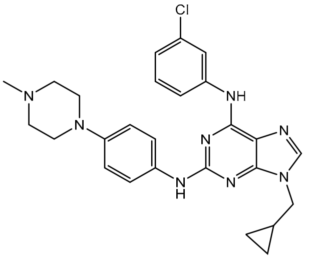

42

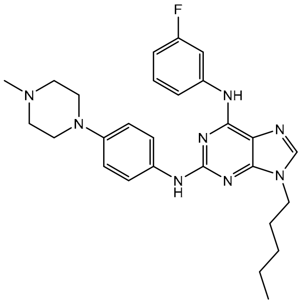

43

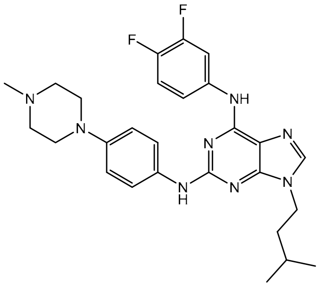

44

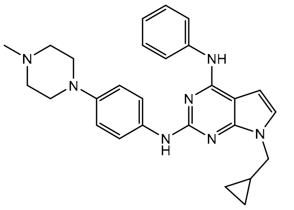

45

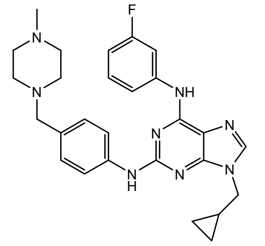

46

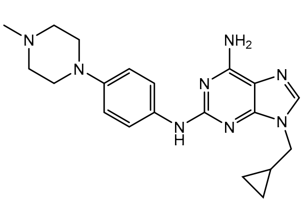

18

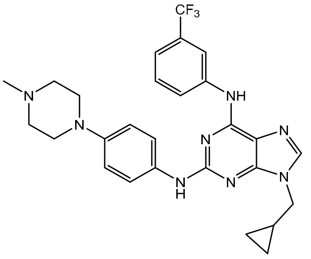

19

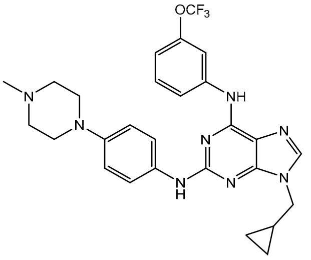

20

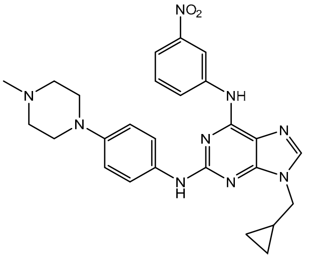

21

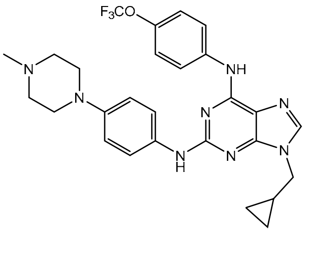

22

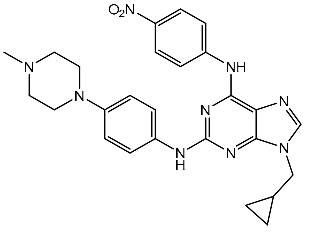

47

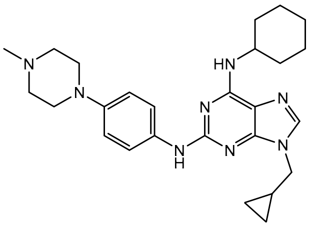

48

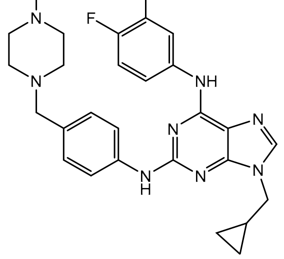

49

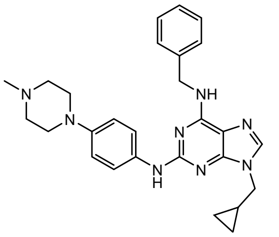

50

51

23

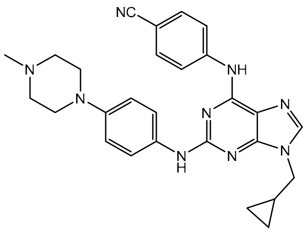

24

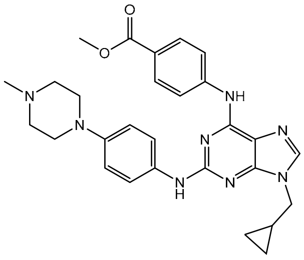

25

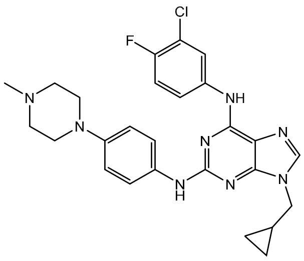

26

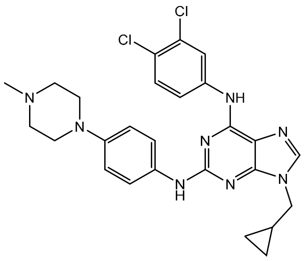

27

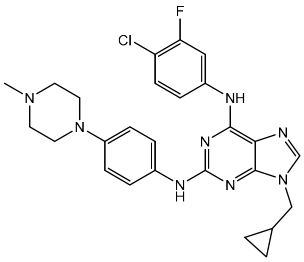

28

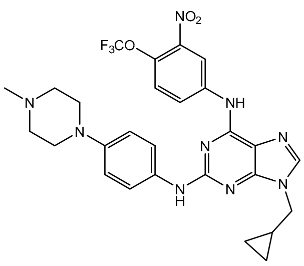

52

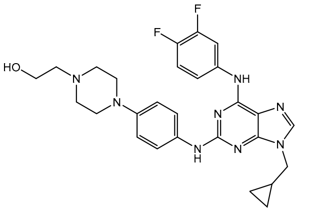

53

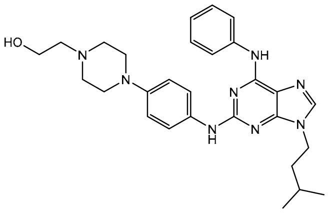

54

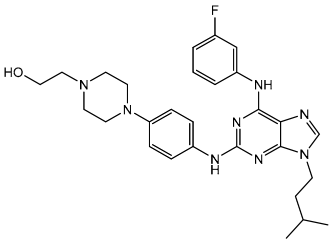

55

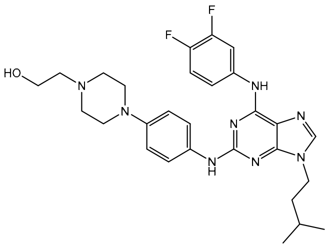

56

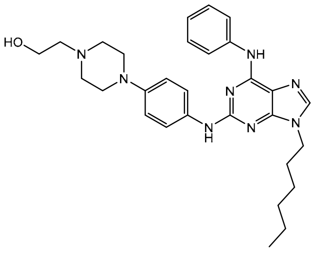

57

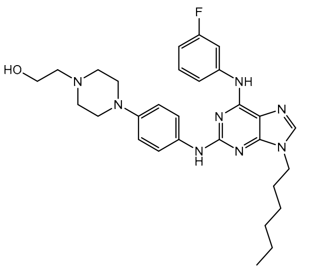

58

29

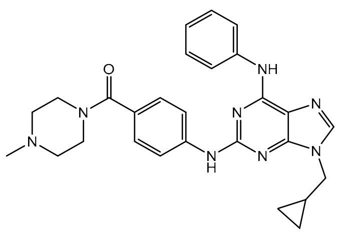

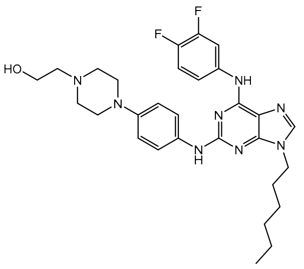

Table S2. Statistical parameters and field combinations for CoMFA and CoMSIA.

Model		q^2	N	SEP	SEE	r^2_{NCV}	F	Field Contributions				
								S	E	H	D	A
**CoMFA-S**		0.477	3	0.676	0.433	0.785	42675	1				
**CoMFA-E**		0.327	13	0.859	0.204	0.966	54659		1			
**CoMFA-SE**		0.576	6	0.637	0.241	0.939	82729	0.587	0.413			
**CoMSIA-S**		0.516	3	0.699	0.499	0.716	29343	1				
**CoMSIA-E**		0.531	8	0.692	0.439	0.811	16100		1			
**CoMSIA-H**		0.44	3	0.700	0.526	0.683	25157			1		
**CoMSIA-D**		0.331	10	0.855	0.805	0.407	1919.00				1	
**CoMSIA-A**		0.002	4	0.948	0.814	0.265	3065.00					1
**CoMSIA-SE**	2,3	0.571	5	0.631	0.329	0.883	50005	0.428	0.572			
**CoMSIA-SEH**	2,3,4	0.637	6	0.589	0.252	0.934	74988	0.249	0.472	0.279		
**CoMSIA-SEHD**	2,3,4,5	0.567	5	0.634	0.31	0.897	57169	0.229	0.404	0.237	0.13	
**CoMSIA-SEHA**	2,3,4,6	0.525	7	0.685	0.285	0.918	49569	0.206	0.381	0.211		0.202
**CoMSIA-SED**	2,3,5	0.569	5	0.612	0.311	0.896	56849	0.337	0.535		0.128	
**CoMSIA-SEA**	2,3,6	0.503	8	0.712	0.295	0.915	40153	0.313	0.476			0.211
**CoMSIA-SEDA**	2,3,5,6	0.514	9	0.716	0.258	0.937	47864	0.281	0.466		0.047	0.206
**CoMSIA-SH**	2,4	0.463	3	0.685	0.505	0.708	28269	0.468		0.532		
**CoMSIA-SD**	2,5	0.481	2	0.665	0.555	0.637	31640	0.757			0.243	
**CoMSIA-SA**	2,6	0.428	6	0.74	0.506	0.732	14574	0.58				0.42
**CoMSIA-SHD**	2,4,5	0.472	2	0.67	0.551	0.643	32401	0.387		0.383	0.231	

**CoMSIA-SHA**	2,4,6	0.424	3	0.71	0.548	0.657	22363	0.305		0.28		0.415
**CoMSIA-SDA**	2,5,6	0.431	6	0.738	0.494	0.745	15607	0.503			0.093	0.404
**CoMSIA-SHDA**	2,4,5,6	0.425	3	0.709	0.546	0.659	22577	0.292		0.269	0.063	0.376
**CoMSIA-EH**	3,4	0.583	6	0.632	0.264	0.927	67984		0.572	0.428		
**CoMSIA-ED**	3,5	0.503	10	0.737	0.278	0.929	36791		0.835		0.165	
**CoMSIA-EA**	3,6	0.245	6	0.87	0.497	0.742	15304		0.658			0.342
**CoMSIA-EHD**	3,4,5	0.608	6	0.612	0.243	0.938	80960		0.52	0.355	0.126	
**CoMSIA-EHA**	3,4,6	0.506	8	0.71	0.263	0.932	51458		0.465	0.312		0.223
**CoMSIA-EDA**	3,5,6	0.252	5	0.833	0.558	0.664	13070		0.551		0.1	0.349
**CoMSIA-EHDA**	3,4,5,6	0.505	8	0.711	0.258	0.935	53519		0.438	0.302	0.052	0.209
**CoMSIA-HD**	4,5	0.457	2	0.68	0.574	0.613	28499			0.74	0.26	
**CoMSIA-HA**	4,6	0.398	5	0.747	0.547	0.678	13883			0.591		0.409
**CoMSIA-HDA**	4,5,6	0.404	5	0.74	0.532	0.695	15017			0.541	0.078	0.381
**CoMSIA-DA**	5,6	0.084	12	1.038	0.626	0.667	4333				0.193	0.807
**CoMSIA-ALL**		0.523	7	0.687	0.279	0.921	51678	0.197	0.364	0.208	0.044	0.186

q^2 = the square of the leave-one-out (LOO) cross-validation (CV) coefficient; N = the optimum number of components; SEP = standard error of prediction; SEE is the standard error of estimation of non-CV analysis; r^2_{ncv} is the square of the non CV coefficient; F is the F-test value; S, E, H, D, and A are the steric, electrostatic, hydrophobic, hydrogen-bond donor, and hydrogen-bond acceptor contributions respectively.

Table S3. Experimental and predicted pIC_{50} and residual values for the studied compounds according to CoMFA and CoMSIA.

		**CoMFA-SE**		**CoMSIA-SEH**				**CoMFA-SE**		**CoMSIA-SEH**	
**Cmpd.**	**pIC_{50} Exp**	**pIC_{50} Pred**	**Res**	**pIC_{50} Pred**	**Res**	**Cmpd.**	**pIC_{50} Exp**	**pIC_{50} Pred**	**Res**	**pIC_{50} Pred**	**Res**
**1**	7.046	7.150	-0.10	7.120	-0.07	**29**	5.917	5.748	0.17	6.066	-0.15
**2**	6.959	7.199*	-0.24	7.089*	-0.13	**30**	6.244	6.010	0.23	6.210	0.03
**3**	7.398	7.464	-0.07	7.509	-0.11	**31**	6.027	5.890	0.14	6.015	0.01
**4**	7.398	7.214	0.18	7.072	0.33	**32**	4.063	3.959	0.10	3.675	0.39
**5**	4.444	6.227**	-1.78	6.239*	-1.80	**34**	4.301	4.346	-0.04	4.567	-0.27

**6**	6.097	6.029	0.07	6.259	-0.16	**35**	5.910	5.821	0.09	5.897	0.01
**7**	6.237	6.289	-0.05	6.388	-0.15	**36**	5.750	5.533	0.22	5.372	0.38
**8**	5.971	6.256*	-0.29	6.047*	-0.08	**37**	5.866	6.443*	-0.58	5.962*	-0.10
**9**	6.222	6.319	-0.10	6.395	-0.17	**38**	5.008	4.952	0.06	5.178	-0.17
**10**	6.237	6.298*	-0.06	6.092*	0.14	**40**	4.469	4.908	-0.44	4.803	-0.33
**11**	6.469	6.476	-0.01	6.331	0.14	**42**	6.187	6.094	0.09	6.045	0.14
**13**	5.074	4.828	0.25	4.850	0.22	**43**	5.642	5.890	-0.25	5.565	0.08
**14**	5.378	5.934	-0.56	6.155	-0.78	**44**	4.206	7.578**	-3.37	7.781*	-3.58
**15**	7.155	6.754	0.40	7.116	0.04	**45**	6.744	6.608	0.14	6.592	0.15
**16**	7.000	6.654	0.35	6.852	0.15	**46**	5.403	7.147**	-1.74	7.229*	-1.83
**17**	6.921	6.915	0.01	6.931	-0.01	**47**	4.956	7.115**	-2.16	6.526*	-1.57
**18**	5.75	6.460	-0.71	5.948	-0.20	**48**	6.649	6.814*	-0.17	6.531*	0.12
**19**	5.939	5.997	-0.06	6.044	-0.10	**49**	6.076	6.194	-0.12	5.929	0.15
**20**	6.770	6.669	0.10	6.782	-0.01	**50**	7.432	7.419*	0.01	7.839*	-0.41
**21**	6.409	7.160**	-0.75	7.206*	-0.80	**51**	7.833	7.909	-0.08	7.724	0.11
**22**	6.886	6.850	0.04	6.709	0.18	**52**	7.703	7.604	0.10	7.792	-0.09
**23**	7.000	6.809*	0.19	6.626*	0.37	**53**	5.907	5.774	0.13	6.234	-0.33
**24**	7.398	7.418	-0.02	7.360	0.04	**54**	6.886	6.820	0.07	6.829	0.06
**25**	6.569	6.839	-0.27	6.663	-0.09	**55**	5.754	5.956*	-0.20	6.079*	-0.32
**26**	7.155	7.030	0.12	6.610	0.54	**56**	6.081	6.404*	-0.32	6.233*	-0.15
**27**	6.699	6.849	-0.15	6.742	-0.04	**57**	6.174	6.193	-0.02	6.069	0.10
**28**	6.357	6.339*	0.02	6.571*	-0.21	**58**	5.775	5.782	-0.01	5.775	0.00

* Test Set compound

** Outliers.

Table S4. q^2 and r^2_{ncv} values after several Y-randomization tests.

	**CoMFA-SE**		**COMSIA-SEH**	
	**q^2**	**r^2ncv**	**q^2**	**r^2ncv**
**Random1**	-0.132	0.23	-0.059	0.116
**Random2**	-0.116	0.225	-0.156	0.164
**Random3**	-0.25	0.276	-0.2	0.101
**Random4**	-0.095	0.185	-0.045	0.132
**Random5**	-0.145	0.194	-0.123	0.117
**Random6**	0.027	0.261	0.087	0.148
**Random7**	-0.176	0.243	-0.196	0.138
**Random8**	-0.071	0.174	-0.053	0.126
**Random9**	-0.034	0.19	-0.065	0.119
**Random10**	-0.299	0.184	-0.215	0.134

^{1}H NMR spectra of compound **7a**.

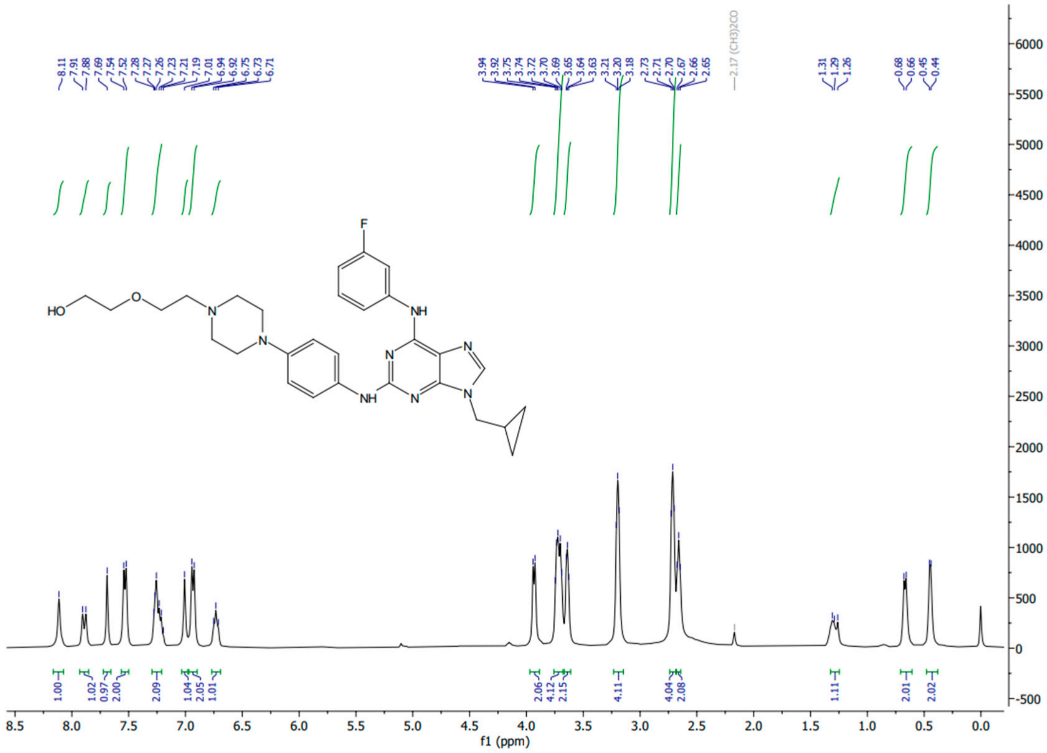

^{13}C NMR spectra of compound **7a**.

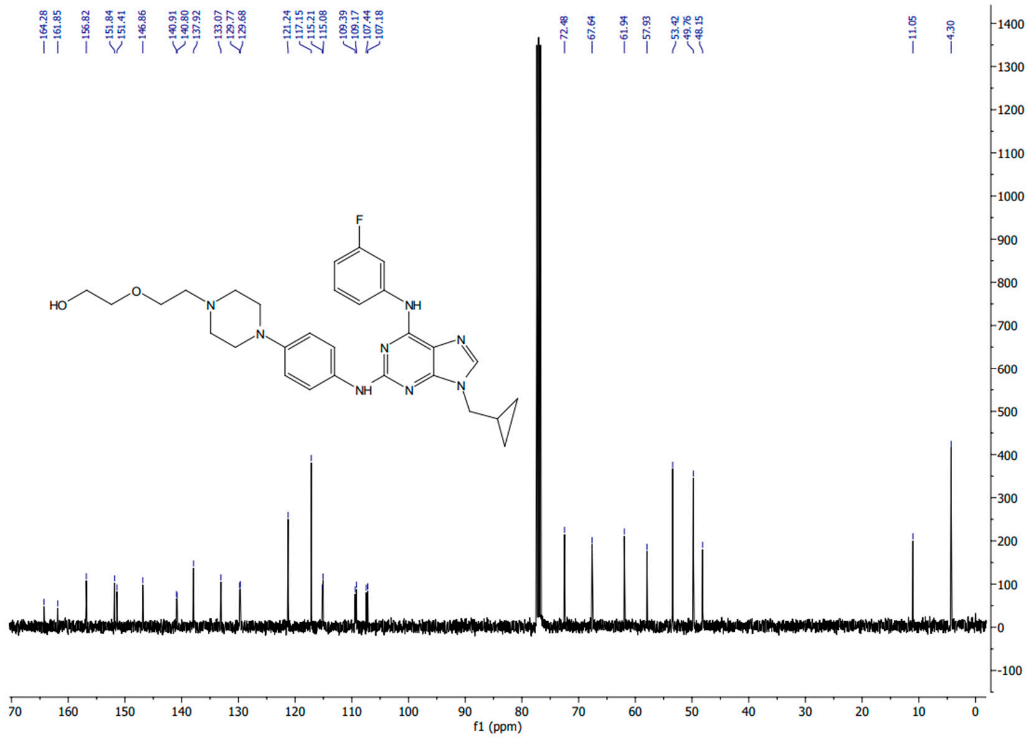

^{1}H NMR spectra of compound **7b**.

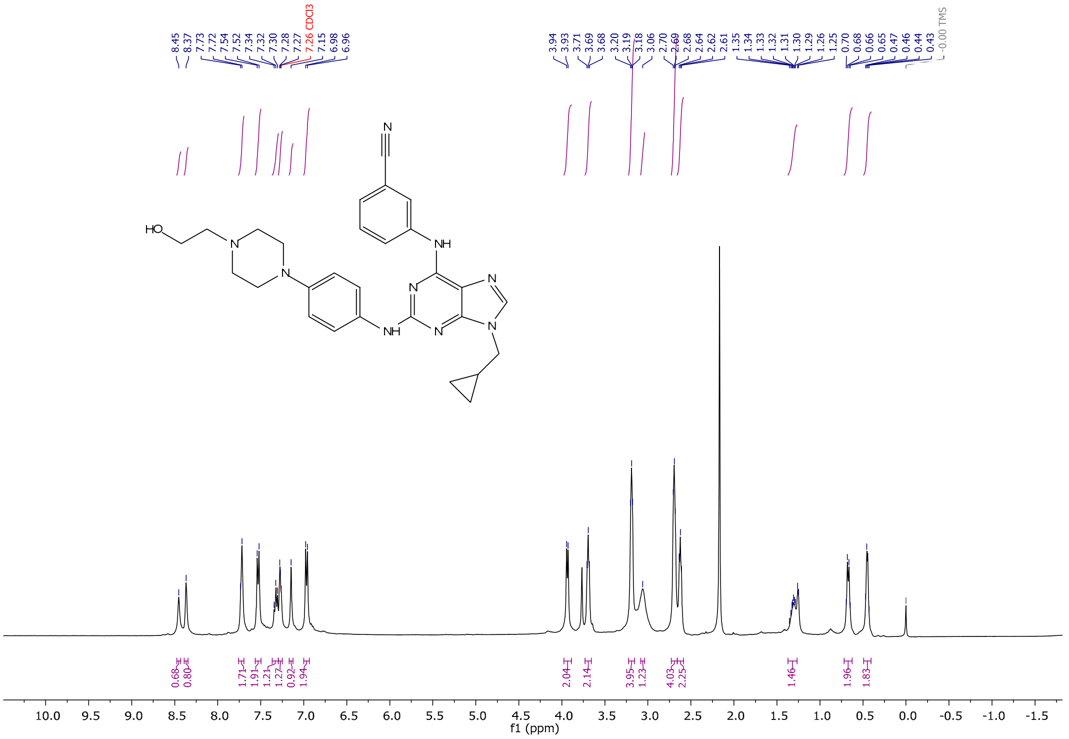

^{13}C NMR spectra of compound **7b**.

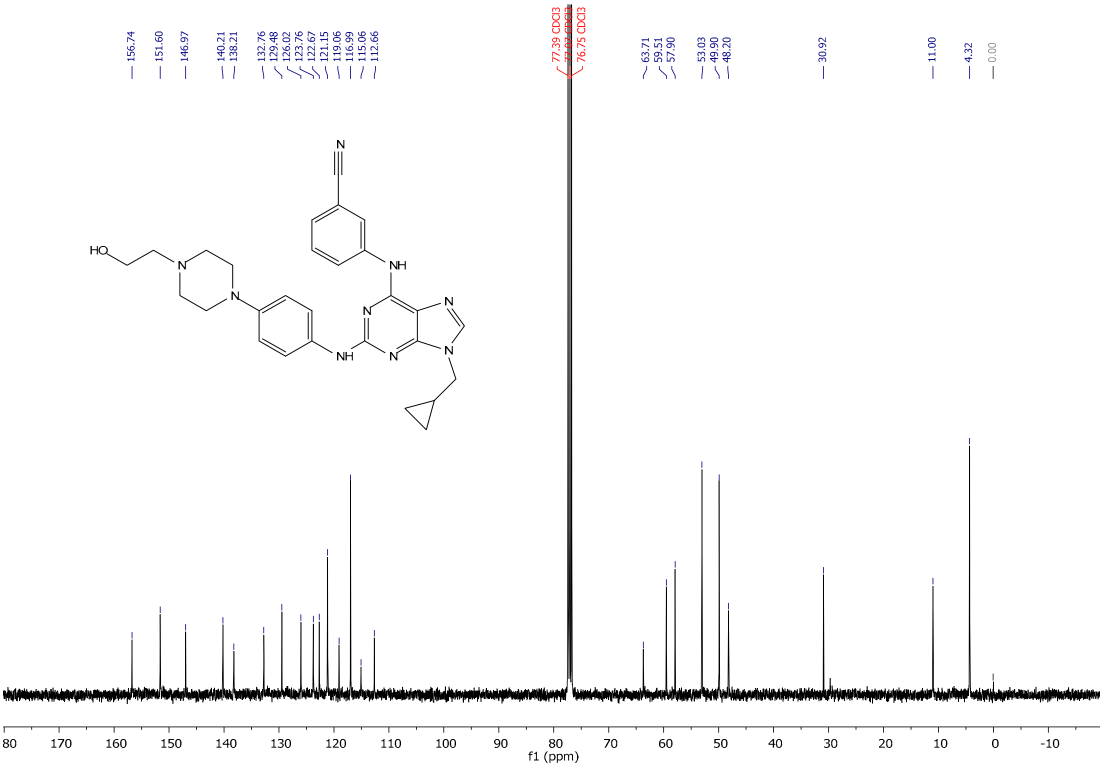

^{1}H NMR spectra of compound **7c**.

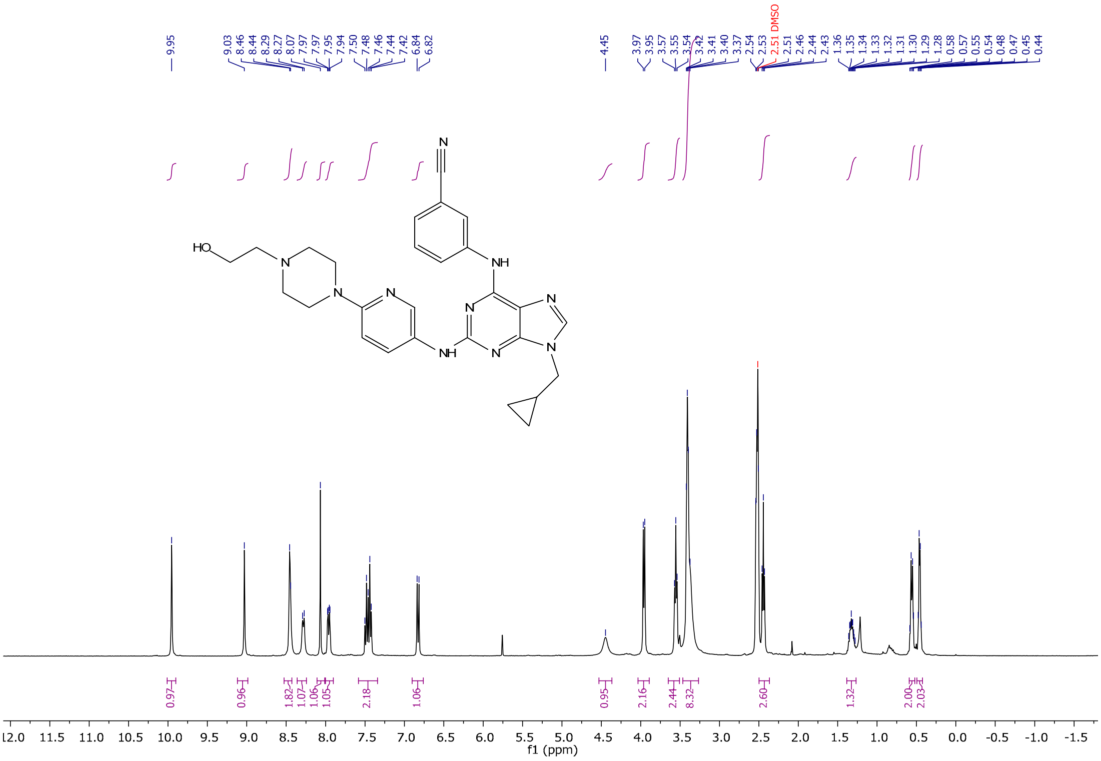

^{13}C NMR spectra of compound **7c**.

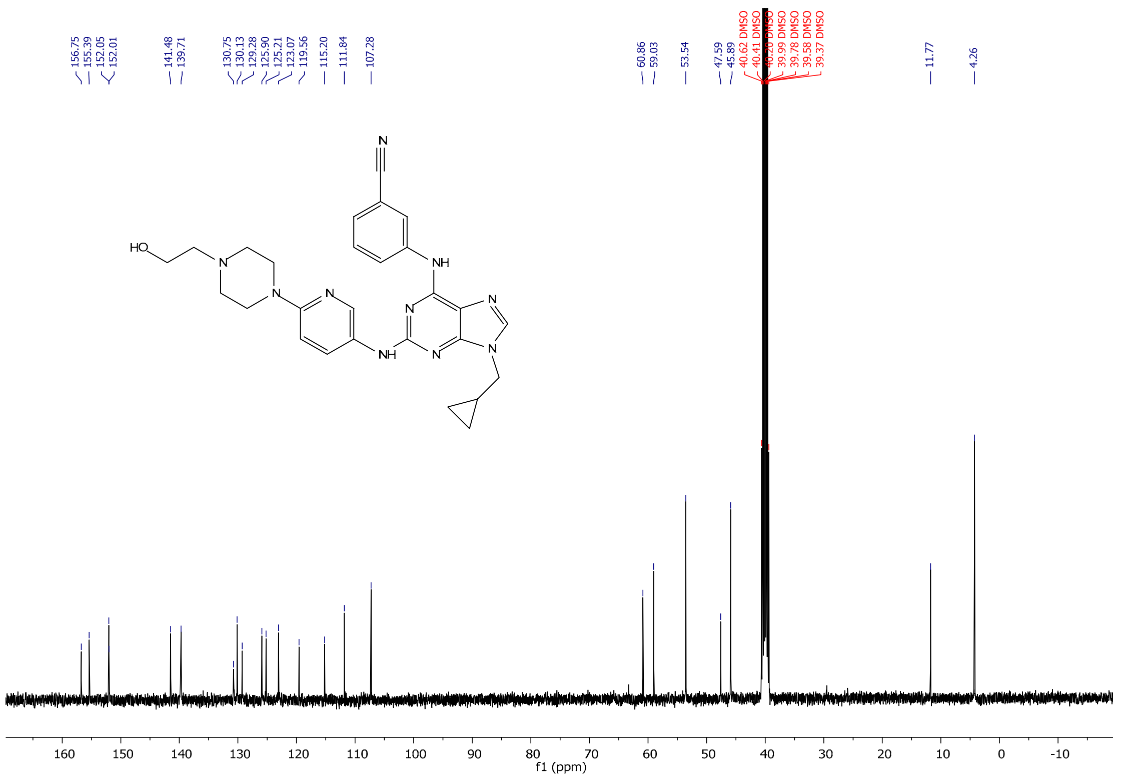

^{1}H NMR spectra of compound **7d**.

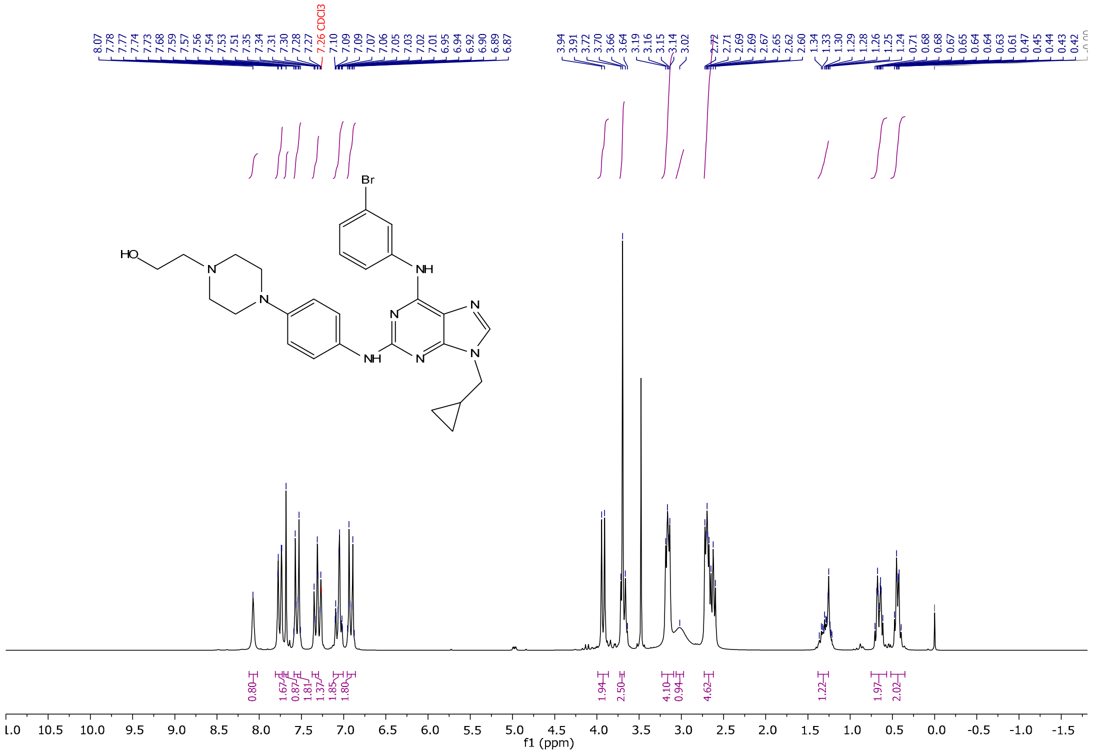

^{13}C NMR spectra of compound **7d**.

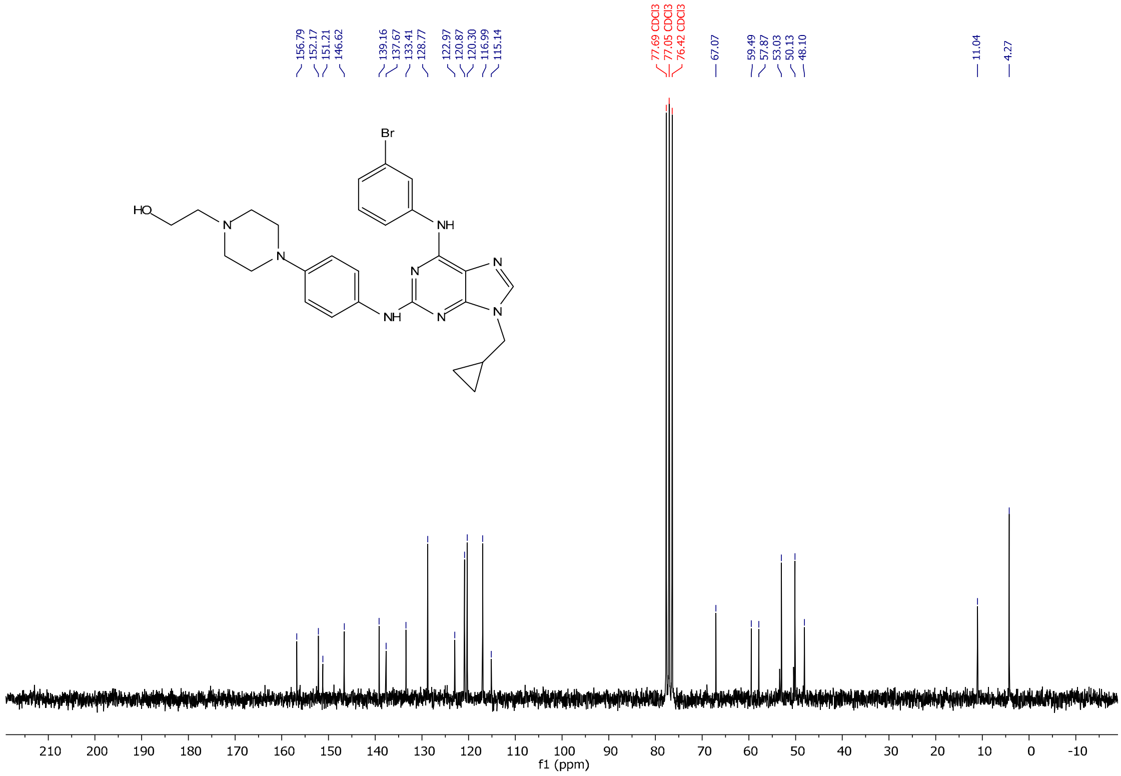

^{1}H NMR spectra of compound **7e**.

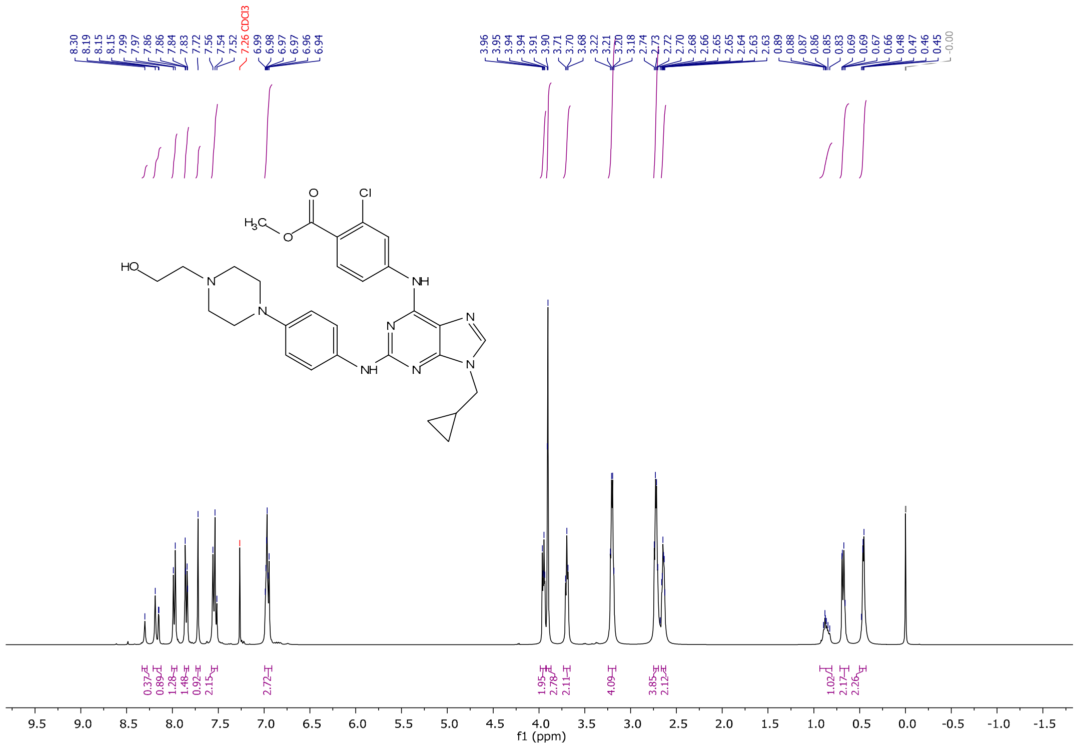

^{13}C NMR spectra of compound **7e**.

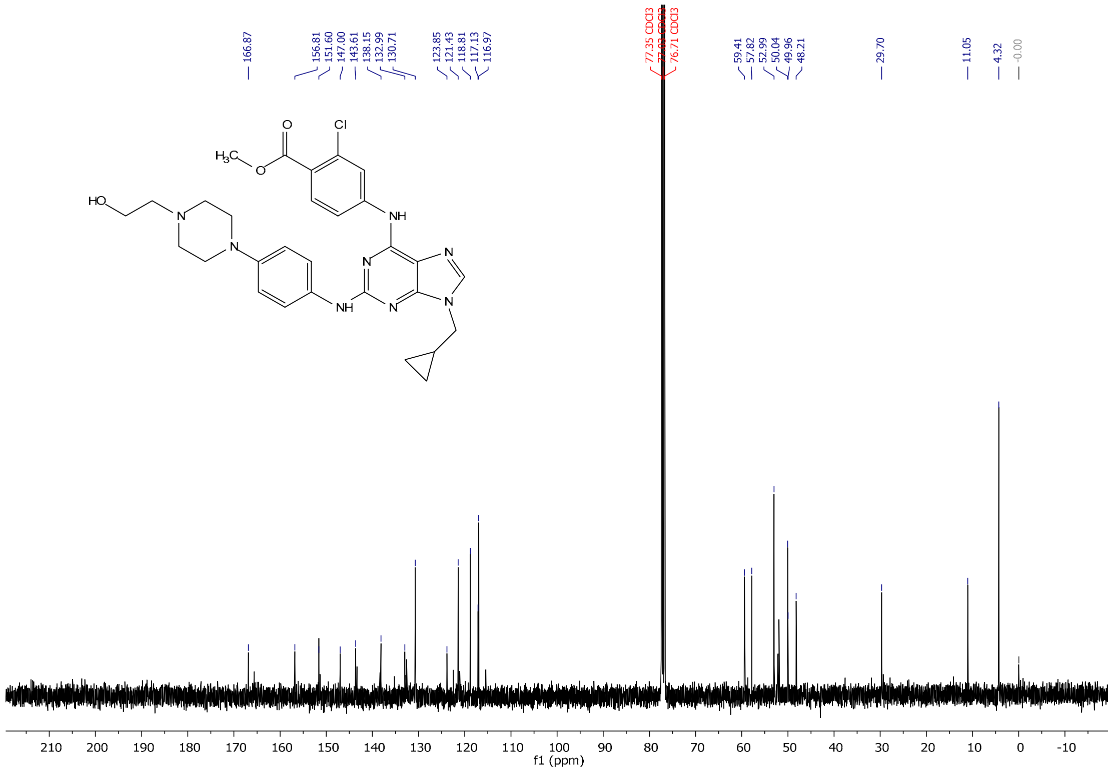

^{1}H NMR spectra of compound **7f**.

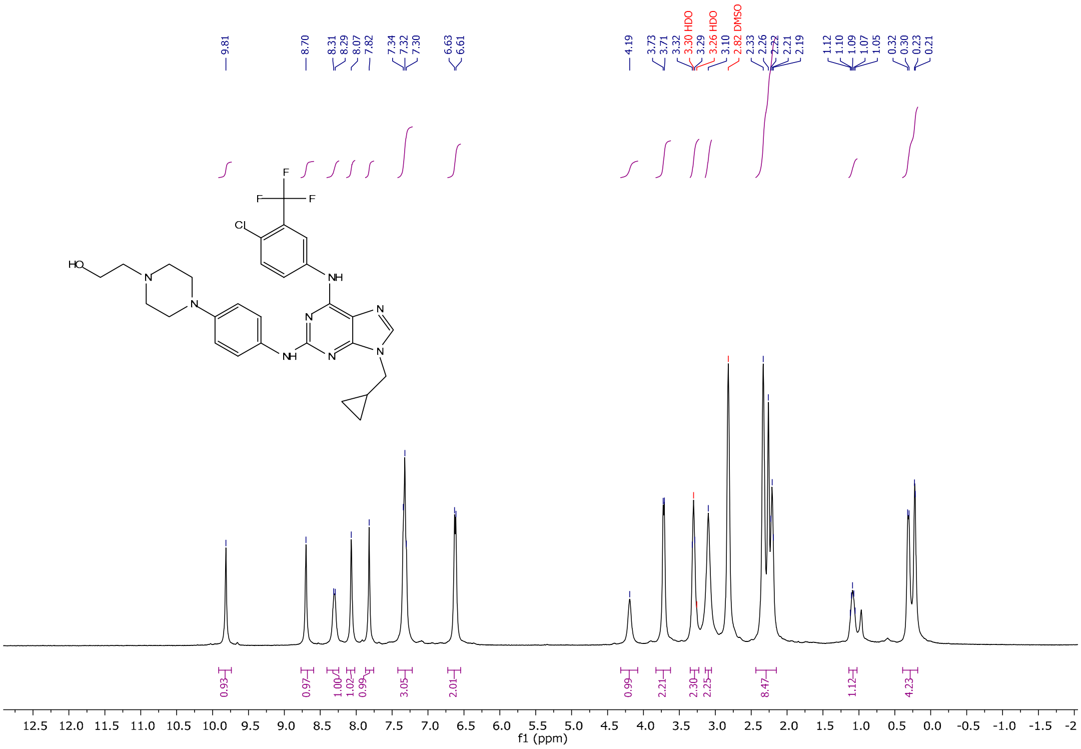

^{13}C NMR spectra of compound **7f**.

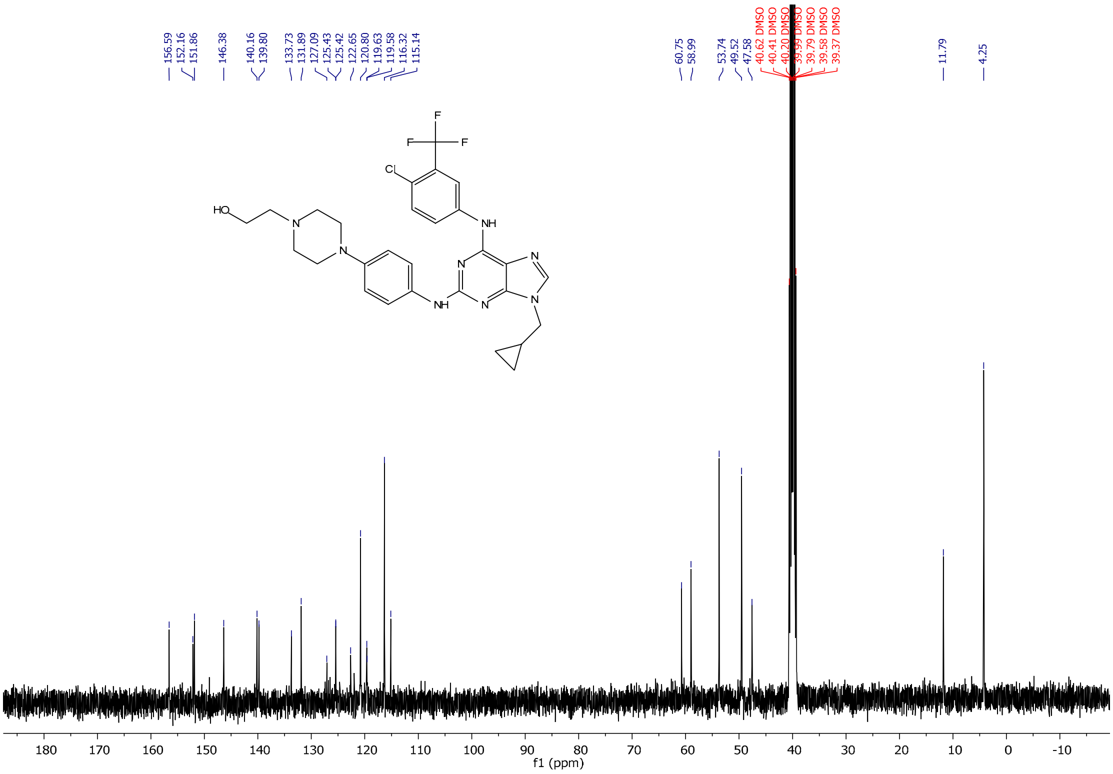

^{1}H NMR spectra of compound **7g**.

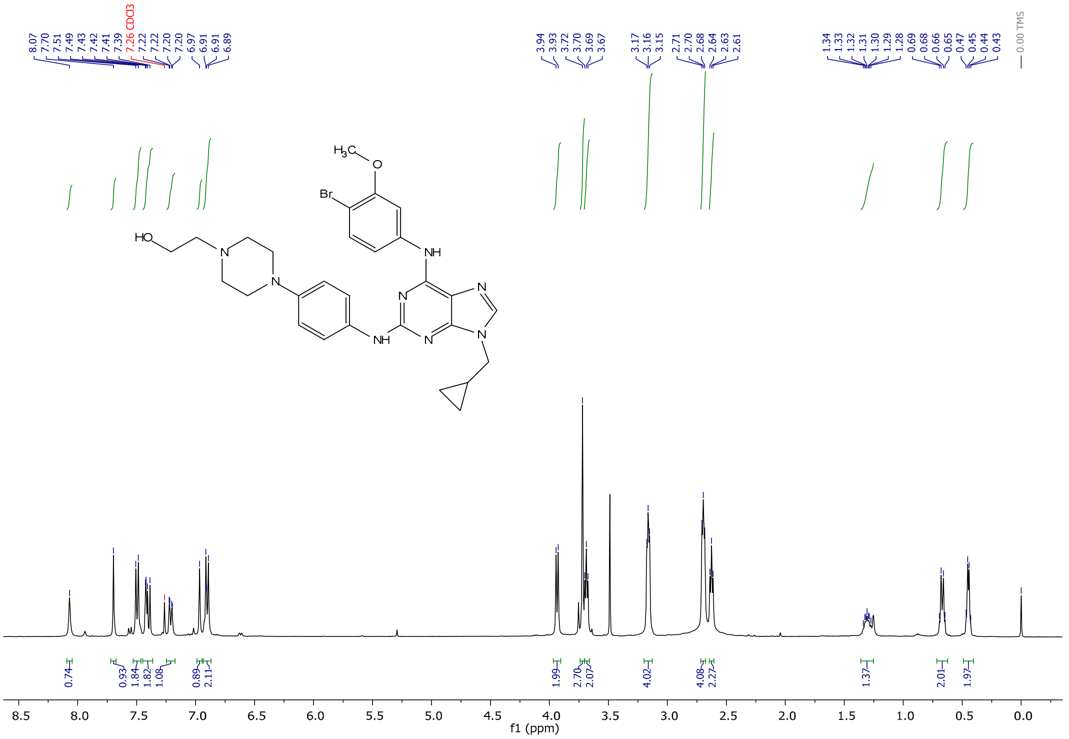

^{13}C NMR spectra of compound **7g**.

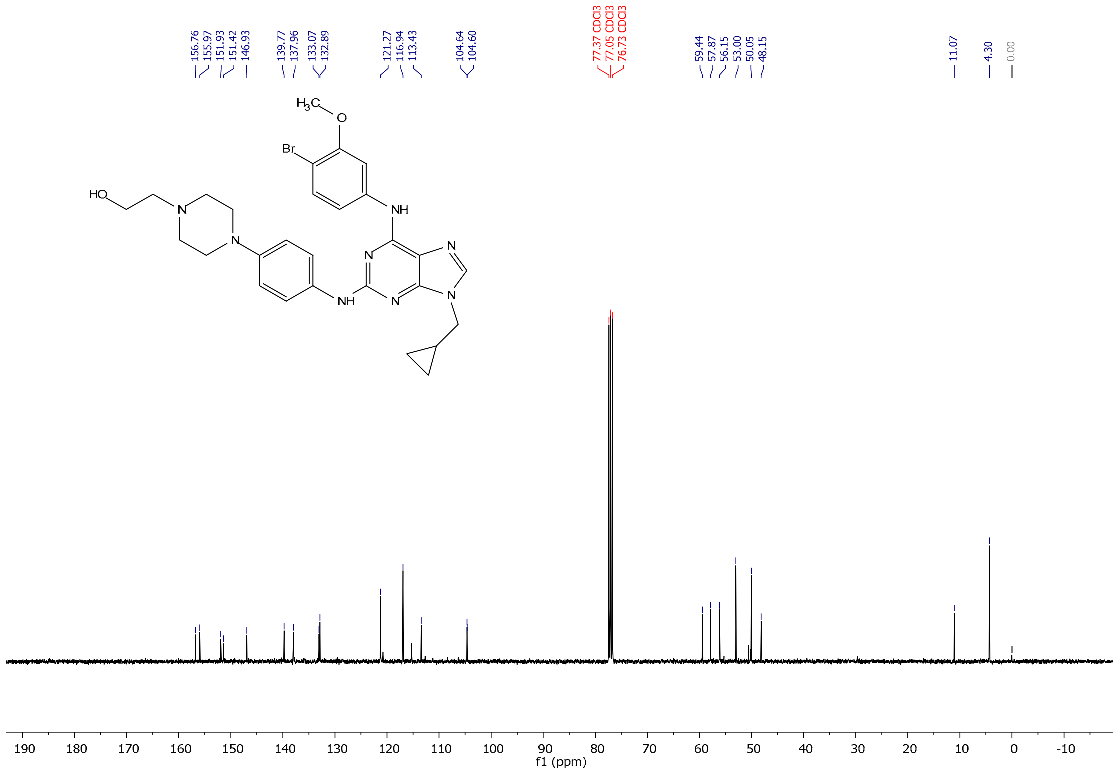

Mass spectra of compound **7a**.

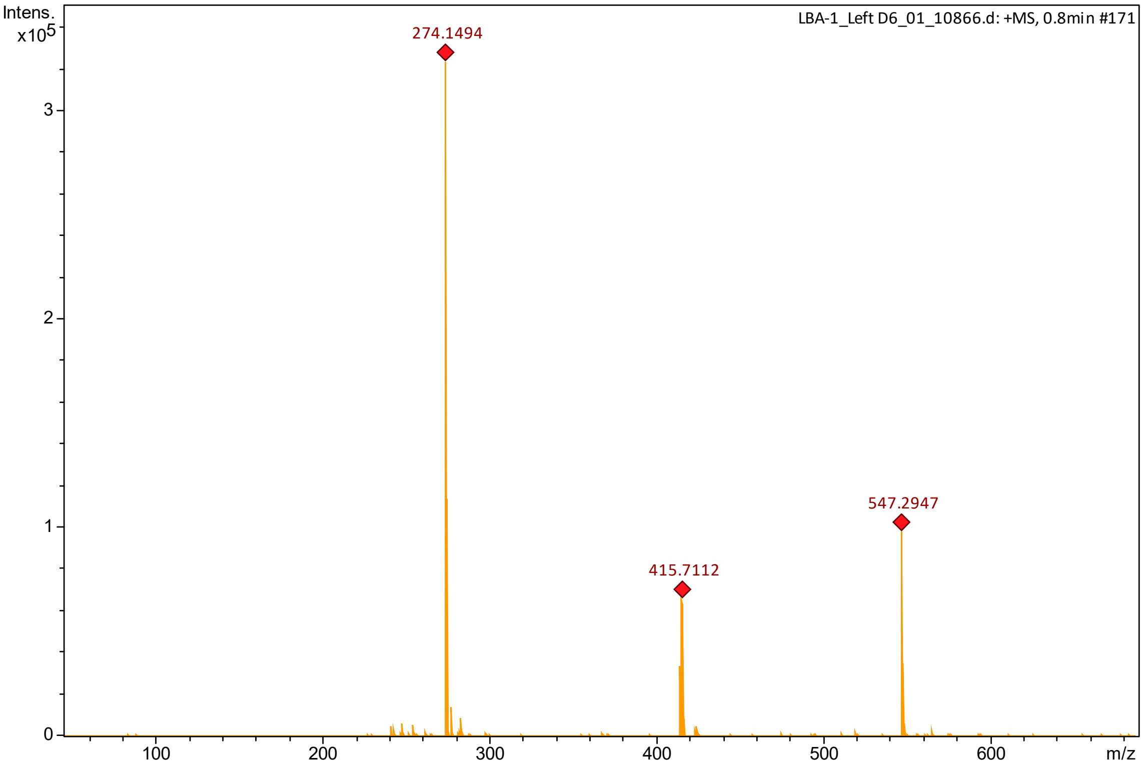

Mass spectra of compound **7b**.

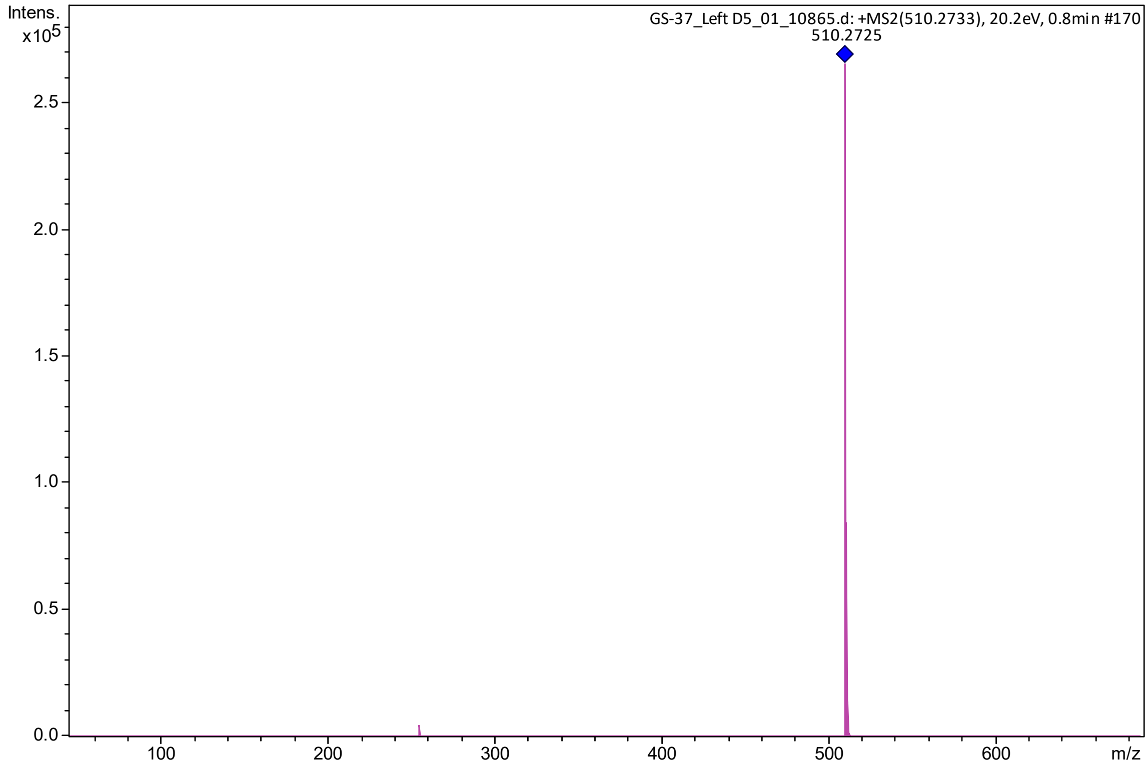

Mass spectra of compound **7c**.

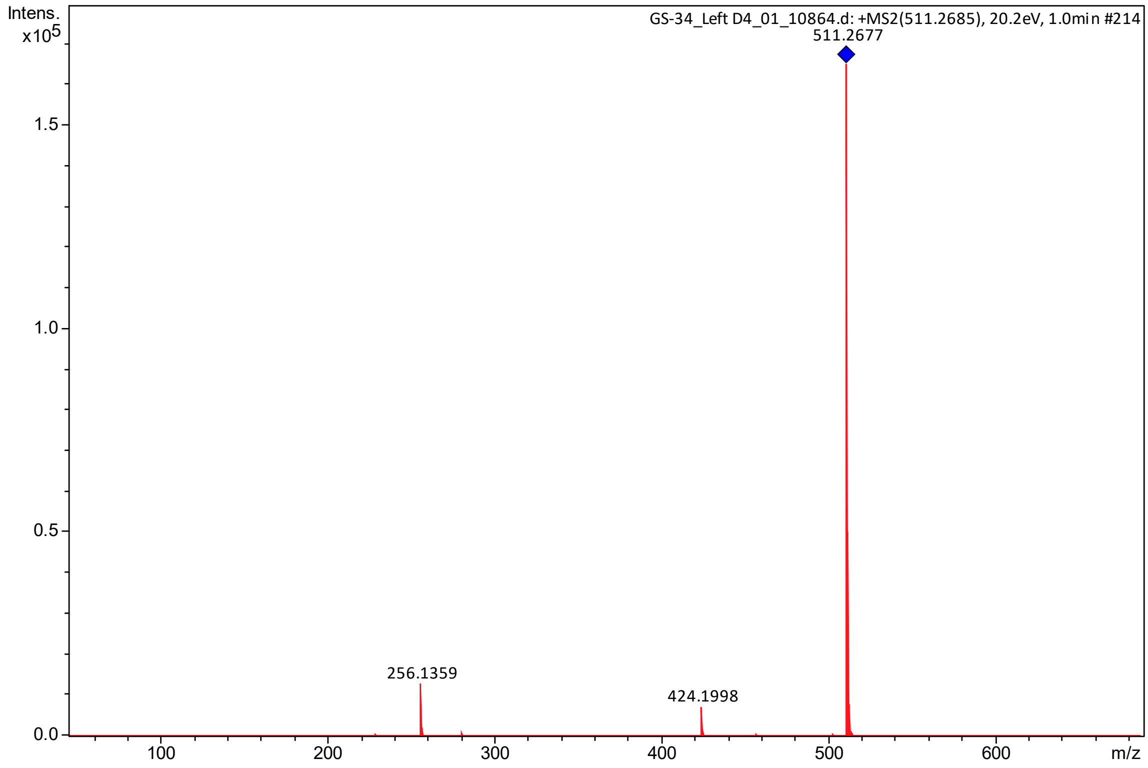

Mass spectra of compound **7d**.

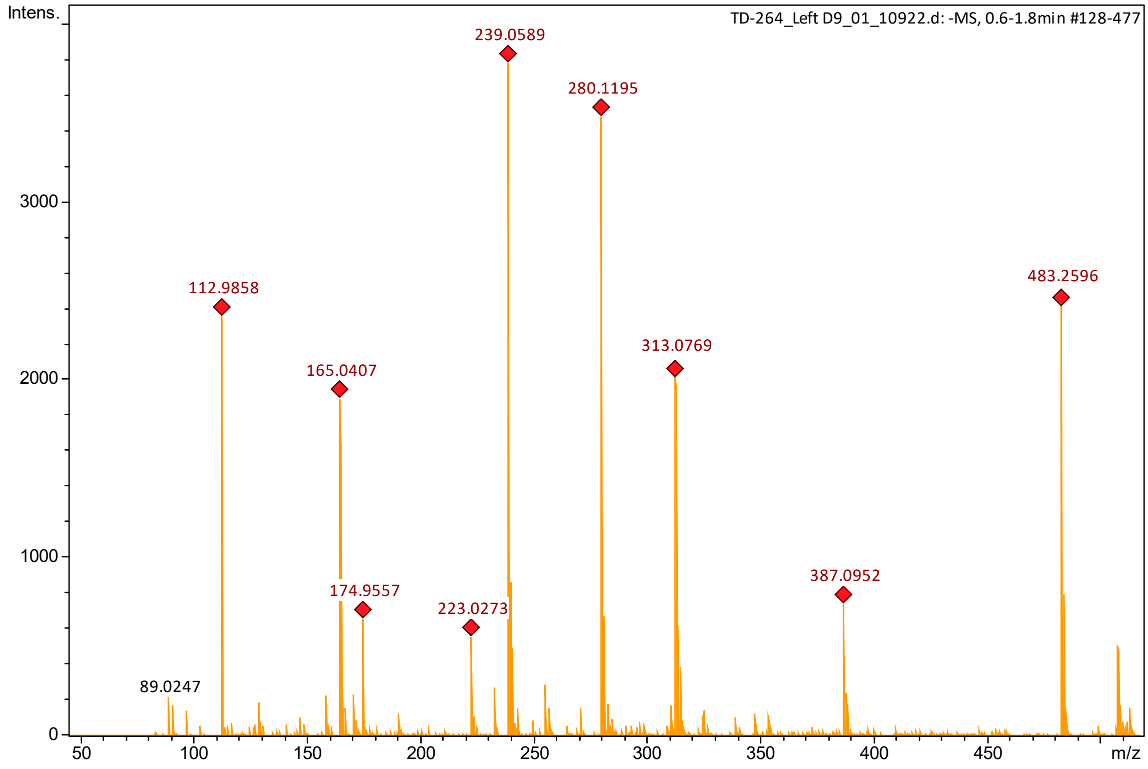

Mass spectra of compound **7e**.

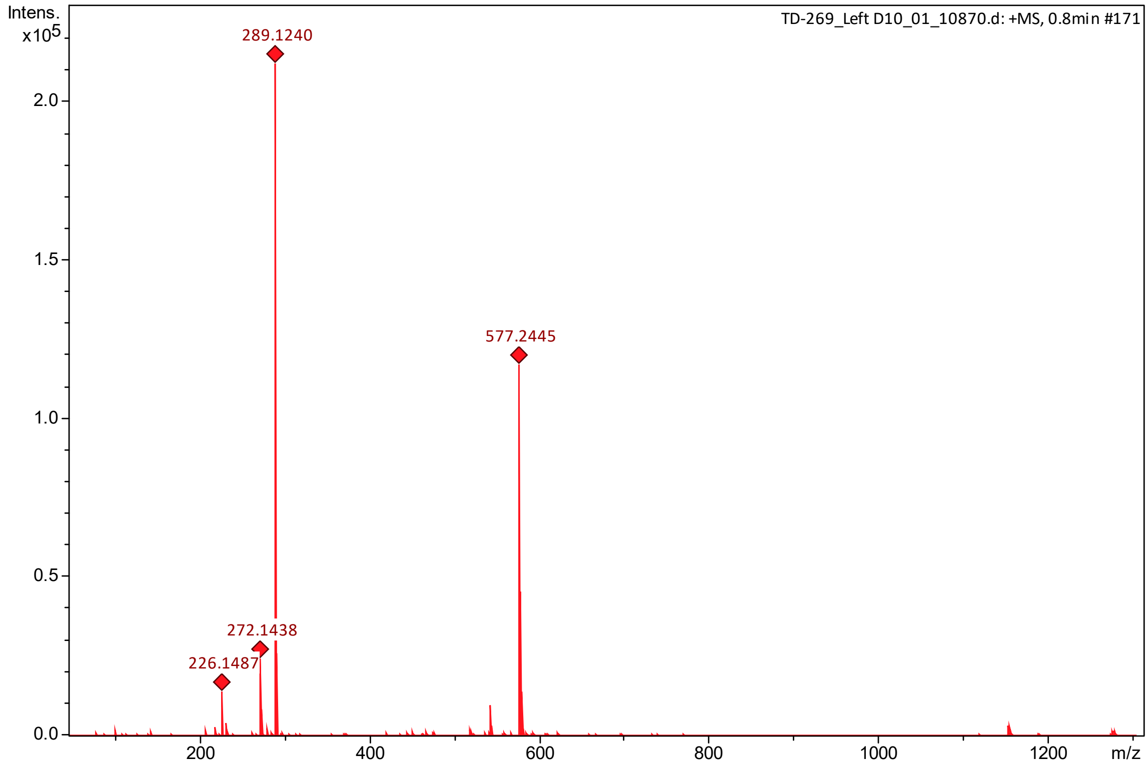

Mass spectra of compound **7f**.

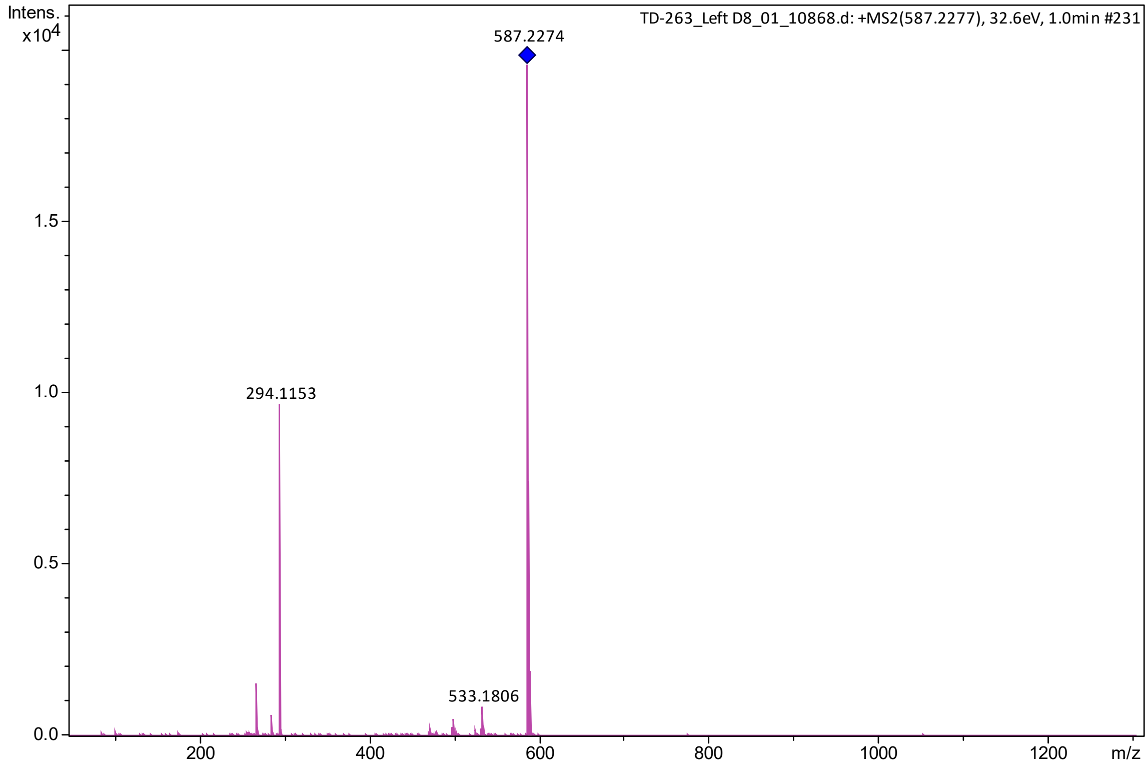

Mass spectra of compound **7g**.

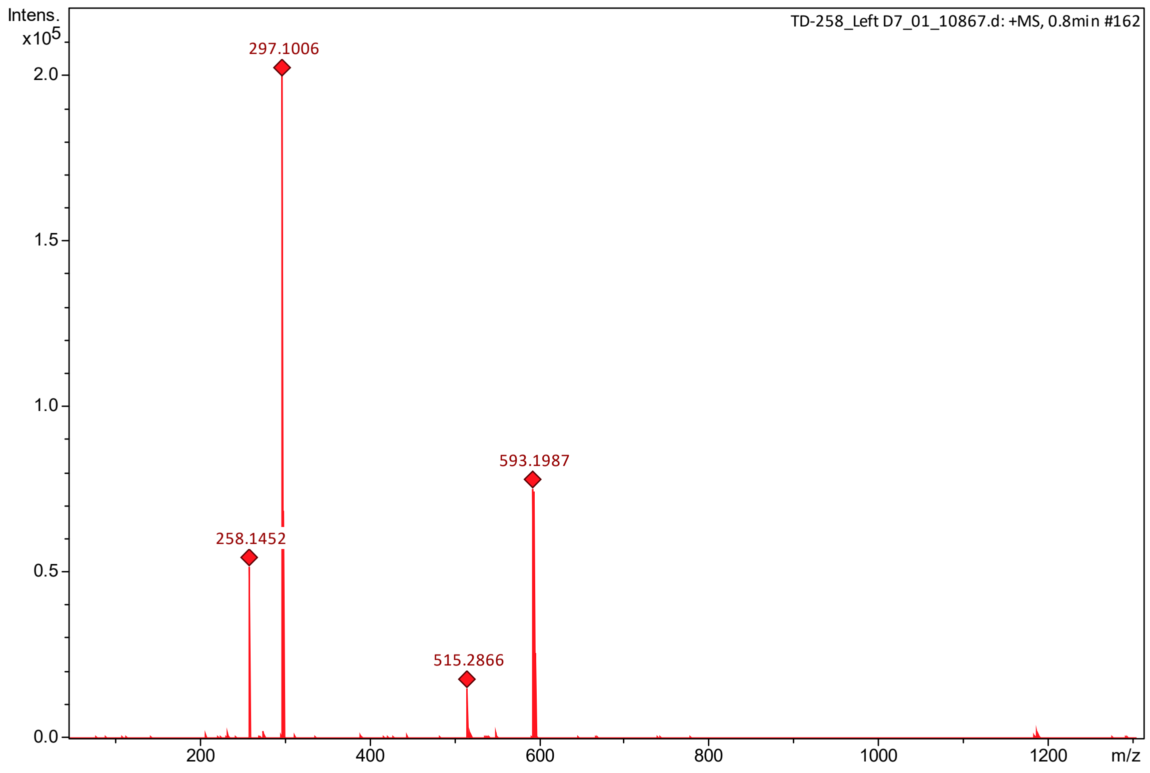

HPLC analysis of compound **7a**.

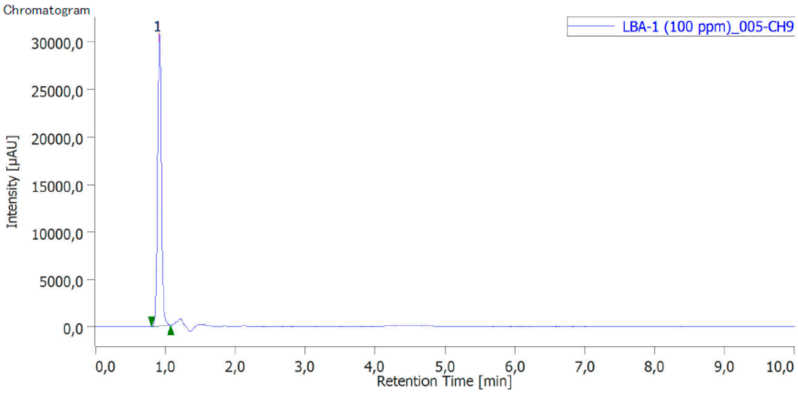

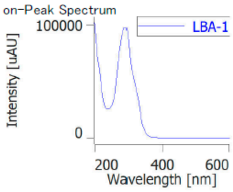

Peak Information

#	Peak Name	CH	tR [min]	Area [μV·sec]	Height [μV]	Area%	Height%	Quantity	Resolution
1	LBA-1	9	0,920	117920	30890	100,000	100,000	N/A	N/A

HPLC analysis of compound **7b**.

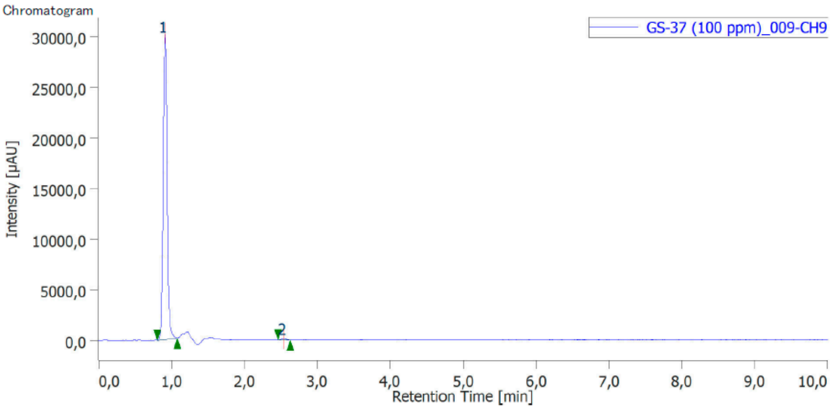

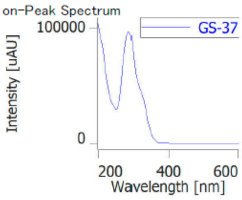

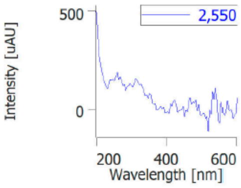

Peak Information

#	Peak Name	CH	tR [min]	Area [μV·sec]	Height [μV]	Area%	Height%	Quantity	Resolution
1	GS-37	9	0,917	114073	30218	99,445	99,562	N/A	14,509
2	Unknown	9	2,550	636	133	0,555	0,438	N/A	N/A

HPLC analysis of compound **7c**.

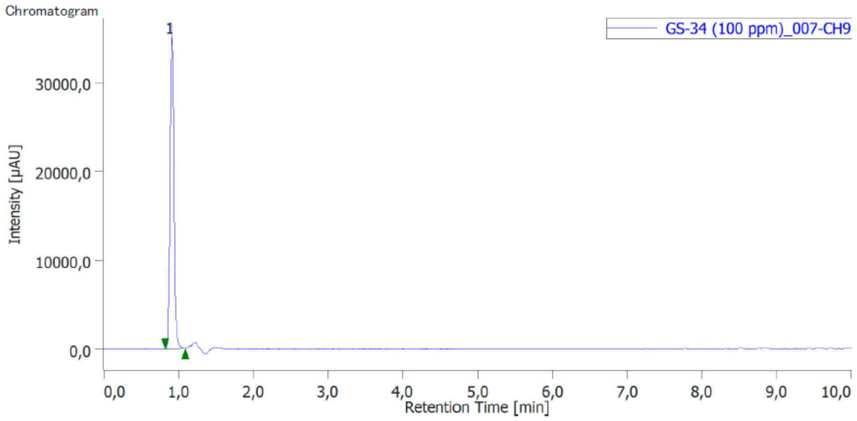

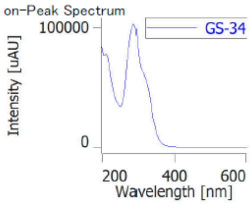

Peak Information

#	Peak Name	CH	tR [min]	Area [µV·sec]	Height [µV]	Area%	Height%	Quantity	Resolution
1	GS-34	9	0,917	129456	35338	100,000	100,000	N/A	N/A

HPLC analysis of compound **7d**.

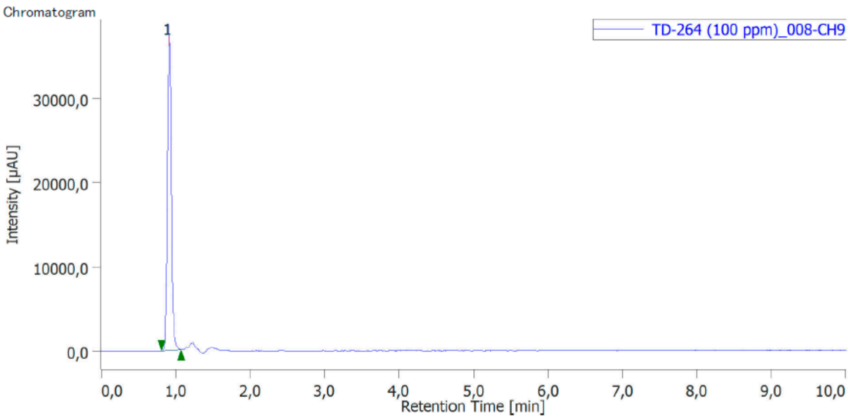

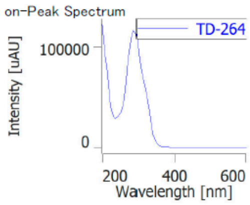

Peak Information

#	Peak Name	CH	tR [min]	Area [µV·sec]	Height [µV]	Area%	Height%	Quantity	Resolution
1	TD-264	9	0,920	139816	37331	100,000	100,000	N/A	N/A

HPLC analysis of compound **7e**.

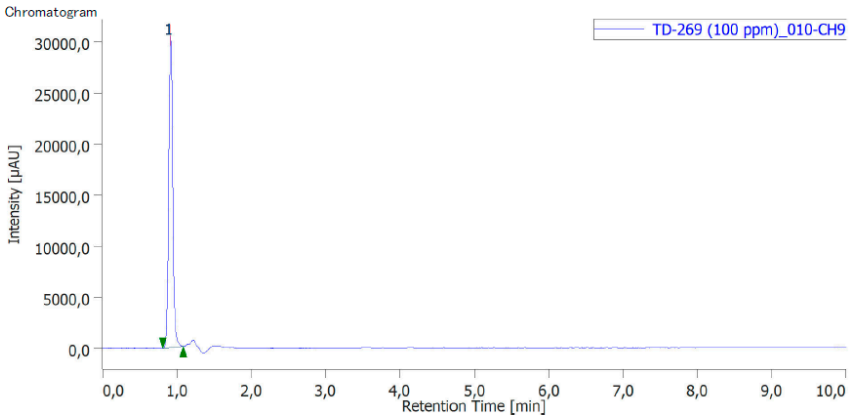

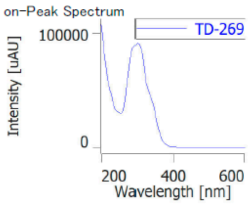

Peak Information

#	Peak Name	CH	tR [min]	Area [µV·sec]	Height [µV]	Area%	Height%	Quantity	Resolution
1	TD-269	9	0,920	117494	30554	100,000	100,000	N/A	N/A

HPLC analysis of compound **7f**.

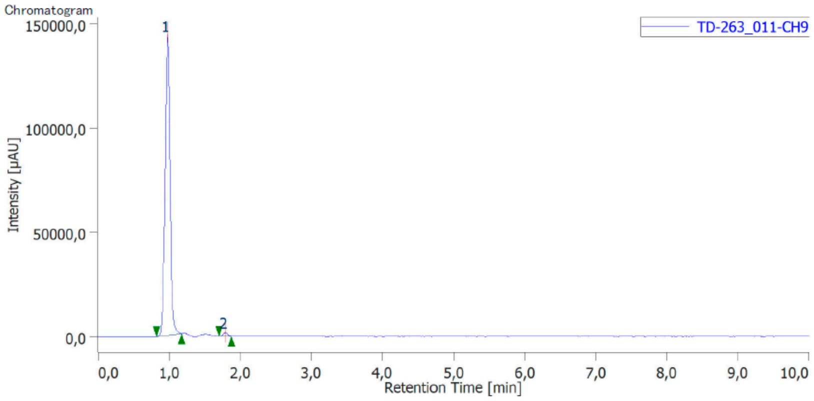

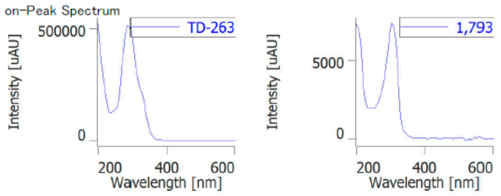

Peak Information

#	Peak Name	CH	tR [min]	Area [µV·sec]	Height [µV]	Area%	Height%	Quantity	Resolution
1	TD-263	9	0,983	701249	144835	98,980	98,797	N/A	6,876
2	Unknown	9	1,793	7229	1764	1,020	1,203	N/A	N/A

HPLC analysis of compound **7g**.

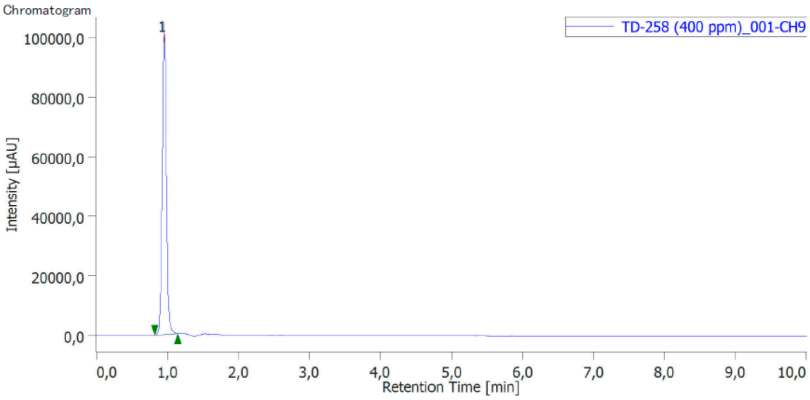

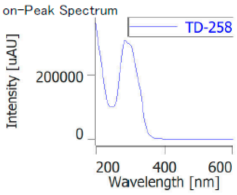

Peak Information

#	Peak Name	CH	tR [min]	Area [µV·sec]	Height [µV]	Area%	Height%	Quantity	Resolution
1	TD-258	9	0,960	420867	101224	100,000	100,000	N/A	N/A

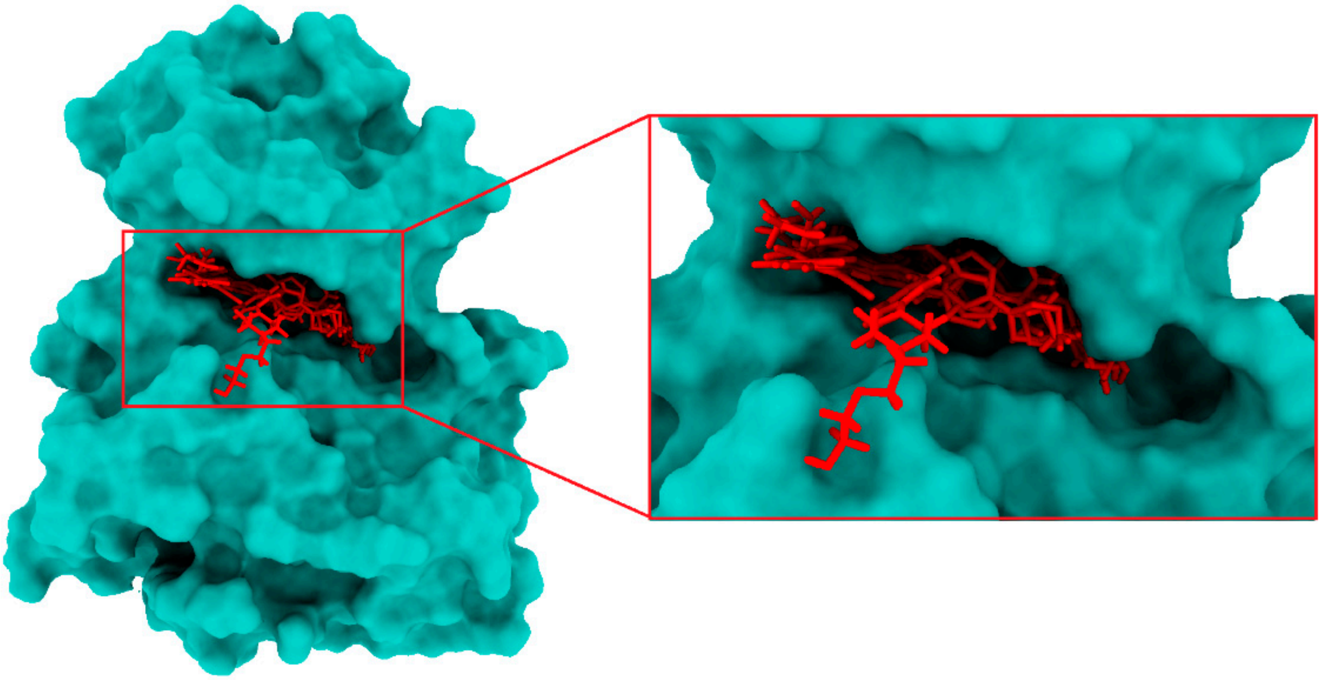

Figure S1. Representation of the Bcr-Abl surface, providing an overview of the binding site location and the conformations adopted by the studied molecules during the docking process (PDB = 6BL8).

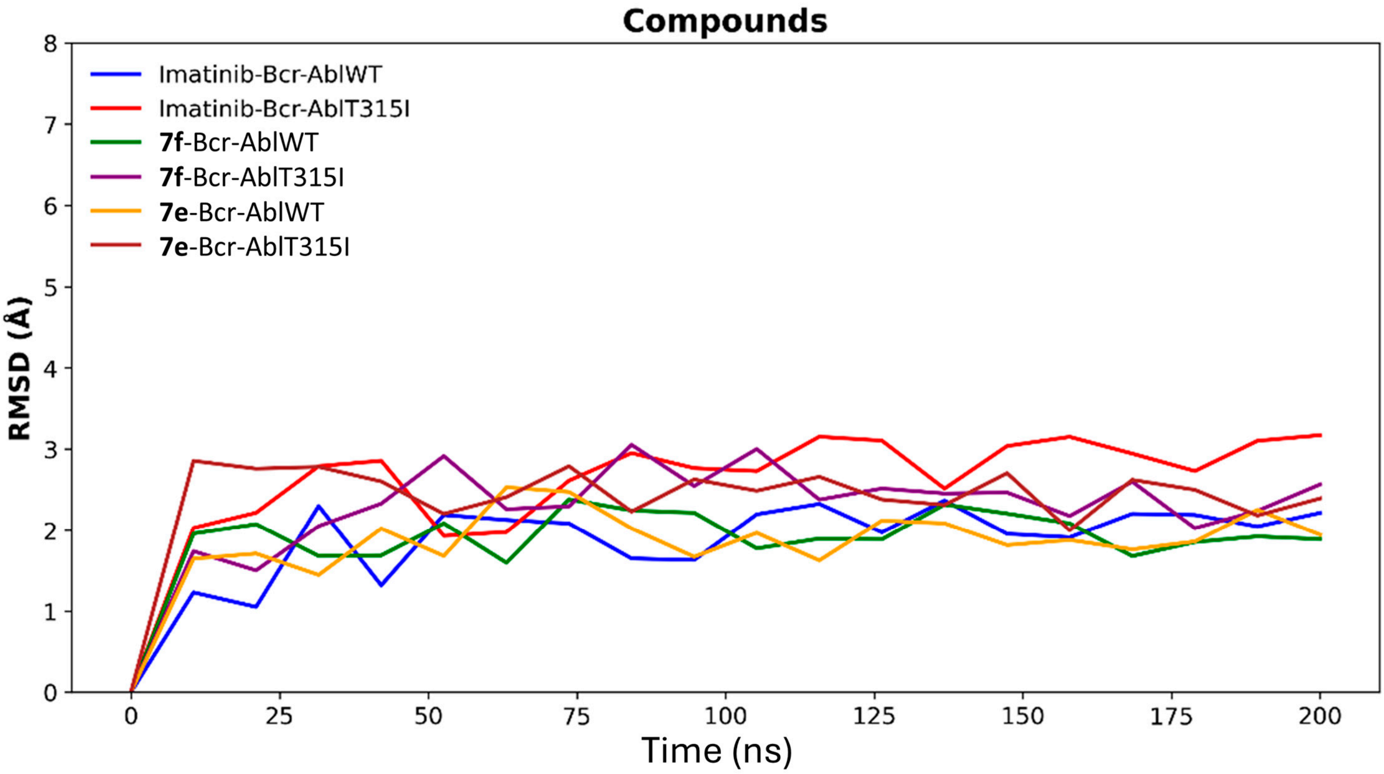

Figure S2. Time evolution of the RMSD for Bcr-Abl complexes with different ligands. Six systems were analysed: imatinib-Bcr-Abl^{WT} (blue), imatinib-Bcr-Abl^{T315I} (red), **7f**-Bcr-Abl^{WT} (green), **7f**-Bcr-Abl^{T315I} (purple), **7e**-Bcr-Abl^{WT} (orange), and **7e**-Bcr-Abl^{T315I} (brown).

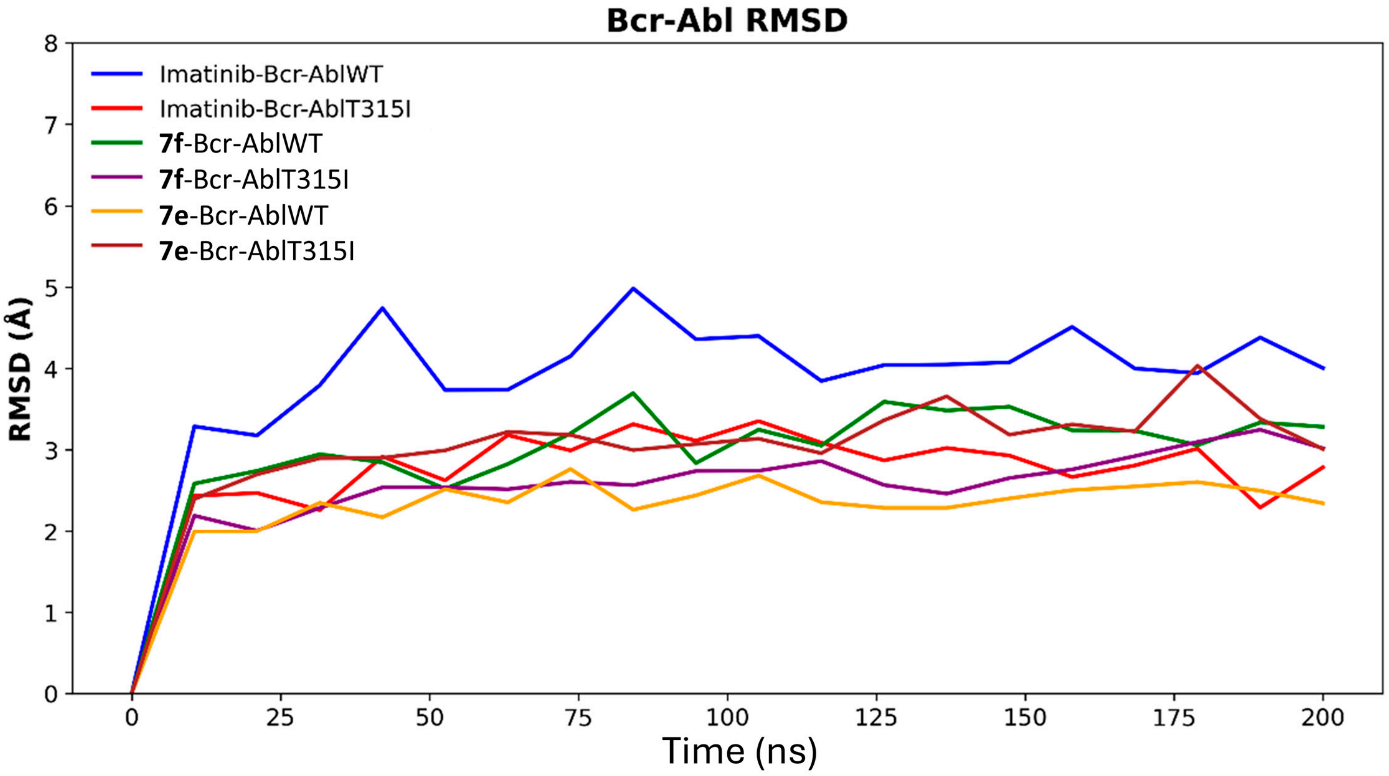

Figure S3. Time evolution of the RMSD for the Bcr-Abl protein in complex with different ligands. Six systems were analysed: imatinib-Bcr-Abl^{WT} (blue), imatinib-Bcr-Abl^{T315I} (red), **7f**-Bcr-Abl^{WT} (green), **7f**-Bcr-Abl^{T315I} (purple), **7e**-Bcr-Abl^{WT} (orange), and **7e**-Bcr-Abl^{T315I} (brown).
